# A *Phytophthora* effector hijacks host StKNOX3‐StHUB1/2 complex to reprogram defense gene expression via histone monoubiquitination

**DOI:** 10.1111/tpj.71112

**Published:** 2026-09-14

**Authors:** Jing Zhou, Xiaoshuang Zhou, Jiahui Nie, Yetong Qi, Lang Liu, Xinya Wu, Yingtao Zuo, Ruimin Yu, Lei Guo, Paul R.J. Birch, Zhendong Tian

**Affiliations:** ^1^ National Key Laboratory for Germplasm Innovation & Utilization of Horticultural Crops Huazhong Agricultural University (HZAU) Wuhan 430070 China; ^2^ Hubei Hongshan Laboratory (HZAU) Wuhan 430070 China; ^3^ Key Laboratory of Potato Biology and Biotechnology (HZAU) Ministry of Agriculture and Rural Affairs Wuhan 430070 China; ^4^ Potato Engineering and Technology Research Center of Hubei Province (HZAU) Wuhan 430070 China; ^5^ Xianghu Laboratory Hangzhou 311231 China; ^6^ Division of Plant Science, School of Life Science University of Dundee (at JHI) Invergowrie, Dundee DD2 5DA UK; ^7^ Cell and Molecular Science James Hutton Institute Invergowrie, Dundee DD2 5DA UK

**Keywords:** class II knotted‐like homeobox (KNOX) transcription factor, effector, StHUB1/2, H2B monoubiquitination, plant immunity, potato late blight

## Abstract

KNOX (KNOTTED1‐like HOMEOBOX) transcription factors regulate the expression of genes involved in plant growth, development, and immunity by specifically binding to DNA elements. Previously, we found that the *Phytophthora infestans* RxLR effector Pi22798 targets the potato transcription factor StKNOX3 to promote colonization. We show here that StKNOX3 interacts with StHUB1 and StHUB2, two E3 ubiquitin ligases that monoubiquitinate histone 2B to form H2Bub1. StKNOX3 stabilizes StHUB1 and StHUB2 in the nucleus, and the StKNOX3‐StHUB1/2 complex acts to enhance *P. infestans* infection. The effector Pi22798 promotes the formation of the StKNOX3‐StHUB1/2 complex, facilitating the nuclear accumulation of StHUB1/2, thereby elevating H2Bub1 levels and increasing plant susceptibility. Combined ChIP‐Seq and RNA‐Seq analyses demonstrate that StKNOX3 modulates transcriptome reprogramming governing cell wall composition, phytohormone responses, and stress responses. StKNOX3 directly binds to the core motifs TGAC or TGTCA in promoter regions of target genes and represses the expression of multiple defense‐related genes. Expression of Pi22798 results in reinforced regulation of the StKNOX3‐targeted genes by affecting the binding affinity of StKNOX3 to their promoters and increasing H2Bub1 levels. Our data support a model in which the host StKNOX3‐StHUB1/2 complex is hijacked by Pi22798 to induce epigenetic modifications that ultimately enhance potato susceptibility to *P. infestans*.

## Significance Statement

Late blight is the most devastating disease of potato, and deciphering how pathogens subvert host defense remains a major challenge. This study reveals that *Phytophthora infestans* suppresses potato immunity by employing effector Pi22798 to hijack a KNOX3 transcription factor–E3 ligase complex, which increases histone H2B monoubiquitination and represses the expression of defense‐related genes, unveiling a pathogen‐driven epigenetic strategy for plant immune suppression.

## INTRODUCTION

Histone ubiquitination is a key epigenetic modification involved in the regulation of gene expression. In eukaryotes, the ubiquitination of histones often occurs at histone H2A and H2B (Mattiroli & Penengo, 2021). The histone 2B monoubiquitination (H2Bub1) pathway involves an enzymatic cascade of E1, E2, and E3 enzymes that sequentially transfer ubiquitin to histone H2B, which has been identified in yeast, Drosophila, mice, humans, and plants (Cao & Ma, 2011; Weake & Workman, 2008). Distinct E3 ubiquitin ligases, Bre1/Bre2 from *Saccharomyces cerevisiae*, RNF20/40 from humans, and HUB1/2 from *Arabidopsis thaliana*, form complexes with specific E2 conjugating enzymes and specifically ubiquitinate the histone H2B at K123, K120, and K143, respectively (Fleury et al., 2007; Kim et al., 2005, 2009; Liu et al., 2007; Wood et al., 2003; Wu et al., 2017). The H2Bub1 modification is involved in epigenetic transcriptional activation or repression (Himanen et al., 2012; Zarreen et al., 2022).

H2Bub1 modification regulates diverse biological processes in plants, including development (e.g., seed germination, flowering) (Bourbousse et al., 2012; Cao et al., 2015; Fleury et al., 2007; Liu et al., 2007) as well as abiotic and biotic stress responses. The *Arabidopsis hub1* mutant shows decreased resistance to the saprophytic fungi *Botrytis cinerea* and *Alternaria brassicicola*, while overexpression of *HUB1* enhances resistance to *B. cinerea*. By contrast, the response of the *hub1* mutant to the bacterial pathogen *Pseudomonas syringae* was unchanged (Dhawan et al., 2009). *Arabidopsis* SNC1 (SUPPRESSOR OF NPR1‐1, CONSTITUTIVE 1) is a key immune regulator involved in the salicylic acid‐dependent plant immunity pathway. H2B monoubiquitination at the disease resistance gene *SNC1* locus regulates its expression and modulates immune responses in *Arabidopsis* (Zou et al., 2014). H2Bub1 is also involved in the immune response of *Arabidopsis* to *Verticillium dahliae* toxins. Compared with wild‐type *Arabidopsis*, *hub1* and *hub2* mutants produce less H_2_O_2_, exhibit reduced cell death, and show impaired activation of defense‐related genes (Zhao et al., 2020). In tomato, silencing of *SlHUB1* and *SlHUB2* reduces the plant's resistance to *B. cinerea*. Silencing of *SlHUB1* enhances resistance to *Pst* DC3000 (Zhang, Li, et al., 2015; Zhang, Liu, et al., 2015), indicating that HUB1 and HUB2 play distinct roles against different pathogens.

KNOX (KNOTTED1‐like HOMEOBOX) transcription factors (TFs) are homeodomain (HD)‐containing proteins that regulate the expression of genes involved in plant growth, development, and stress responses (Hay & Tsiantis, 2010). *KNOX* genes are divided into three classes based on their sequence similarity, expression pattern and functions (Magnani & Hake, 2008). Class II KNOX TFs contain four genes in *Arabidopsis*: *KNAT3*, *KNAT4*, *KNAT5*, and *KNAT7*, which can be subgrouped into the *KNAT3/4/5* and *KNAT7* clades (Mukherjee et al., 2009). The well‐characterized *KNAT3* is involved in embryo sac development, integument and ovule formation, flower formation, ABA‐induced seed germination and early development, root nodule development, grain size, and bolting time (Azarakhsh et al., 2015, 2020; Chen et al., 2023, 2025; Di Giacomo et al., 2017; Hackbusch et al., 2005; Kim et al., 2013; Pagnussat et al., 2007; Sheng et al., 2022). Recently, KNAT3 together with another KNOX TF, KNAT7, has been reported to show overlapping roles and partial functional redundancy in xylem vessel development during secondary cell wall (SCW) formation and seed coat mucilage biosynthesis (Qin et al., 2020; Wang et al., 2020; Zhang et al., 2022). Besides regulating plant growth process, class II *KNOX* genes also function in biotic stress responses. Potato StKNOX3 enhances susceptibility to an oomycete pathogen *Phytophthora infestans* (Zhou et al., 2022). HOS59, an orthologue of KNAT3, suppresses the NLR immune receptor BRG8‐mediated immunity in rice (Xu et al., 2024). However, how these class II KNOX TFs suppress plant immunity remains unknown.

Late blight remains the most devastating potato disease, threatening potato production and quality. As the causal agent of late blight, *P. infestan*s secretes various effector proteins into the plant cell apoplast or host cells during infection (Wang et al., 2025). Current studies reveal that effectors mainly regulate plant immune responses by alteration of host protein stability, target relocalization, disruption of protein complexes, and modulation or utilization of host enzyme activities (Fabro, 2022; He et al., 2020; Wang et al., 2023). Plant immune responses: PTI and ETI are significantly overlapping at the transcriptional level (Ding et al., 2020). Given the importance of transcription in regulating plant immunity, it is unsurprising that approximately 30% of host proteins targeted by effectors are involved in this process (He et al., 2020). Several oomycete effectors directly target host TFs to repress defense gene activation or upregulate susceptibility genes. For instance, *P. infestans* effector AVR2 upregulates the potato TF CHL1 to induce brassinosteroid (BR)‐marker gene expression and enhance susceptibility to *P. infestans* (Turnbull et al., 2017). CRN12_997 from *Phytophthora capsici* binds the TCP transcription factor, SlTCP14‐2, to diminish its DNA binding to the promoters of the immune regulatory, thus enhancing susceptibility to *P. capsica* (Stam et al., 2021). A nucleus‐localized RxLR effector PsAvh110 from the pathogen *Phytophthora sojae* disrupts the assembly of the GmLHP1‐2/GmPHD6 transcriptional complex and represses the expression of a subset of immune‐associated genes to modulate plant immunity (Qiu et al., 2023). Interestingly, effectors can also manipulate defense gene expression by interfering with epigenetic modifications. An effector protein from the arbuscular mycorrhizal fungus *Rhizophagus irregularis* interacts with host histone H2B protein and reduces the H2Bub1 levels, thus downregulating defense‐related genes and promoting mycorrhizal colonization (Wang et al., 2021). The cytoplasmic effector PsAvh23 of *P. sojae* binds to a subunit of the histone acetyltransferase (HAT) complex and disrupts its assembly with the catalytic subunit GCN5, thereby suppressing H3K9 acetylation and dysregulating defense‐related genes, thus increasing plant susceptibility (Kong et al., 2017).

Our previous work revealed that the *P. infestans* effector Pi22798 targets StKNOX3 and StKAKU4 to enhance plant susceptibility, respectively (Zhou et al., 2022, 2026). However, the target genes regulated by StKNOX3, how these genes contribute to susceptibility, and the mechanism by which effector Pi22798 modulates plant defense through StKNOX3 remain undetermined. Here, we show that StKNOX3 interacts with StHUB1 and StHUB2, two E3 ubiquitin ligases that monoubiquitinate histone H2B, forming H2Bub1. Pi22798 facilitates assembly of the StKNOX3‐StHUB1/2 complex, thereby increasing the protein stability of StHUB1/2 in the nucleus and enhancing H2Bub1 levels. Pi22798 promotes homodimerization of StKNOX3 and its subsequent association with StHUB1/2 in the nucleus. This leads to elevated H2Bub1 levels, which in turn enhance the ability of StKNOX3 to repress defense‐related genes, thereby compromising plant immunity. This study highlights a counter‐defense mechanism by which a pathogen effector suppresses immunity by co‐opting the StKNOX3‐StHUB1/2 module to inhibit defense gene activation.

## RESULTS

### KNOX3 interacts with the H2B monoubiquitination complex HUB1/2

**Figure 1:**
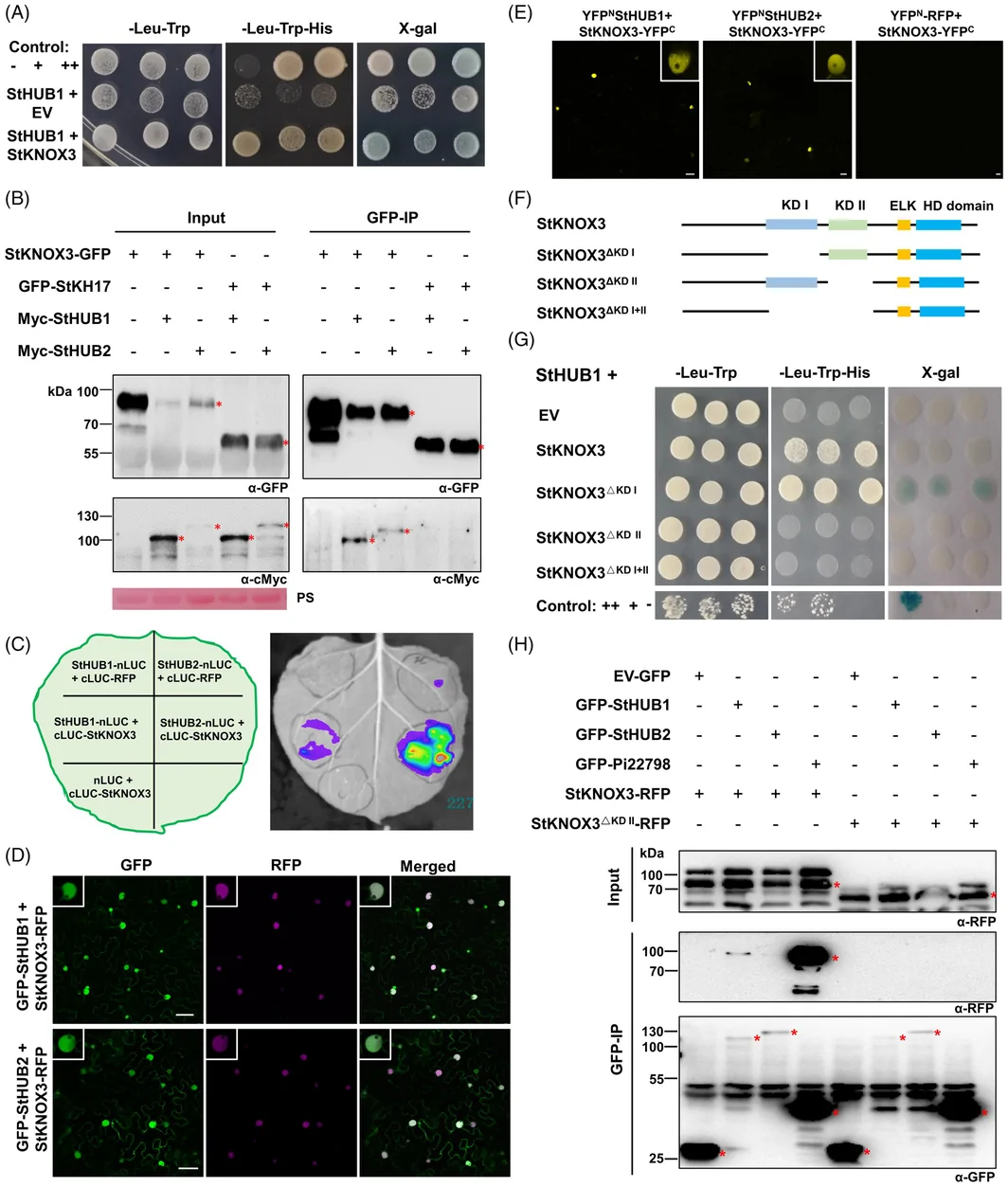
StKNOX3 interacts with the H2B monoubiquitination proteins StHUB1 and StHUB2. (A) Y2H assay showing the interaction between StKNOX3 and StHUB1. Yeast co‐expressing pDest32‐StHUB1 with pDest22‐StKNOX3 grew on selective medium SD/‐Trp/Leu/‐His and yielded *β*‐galactosidase (X‐Gal) activity, but did not when co‐expressed with the empty control. (B) Co‐IP assay showing that StKNOX3 interacts with StHUB1 and StHUB2 *in vivo*. Total protein was extracted from infiltrated *Nicotiana benthamiana* leaves and IP was performed with GFP beads followed by immunoblotting with anti‐GFP or anti‐cMyc antibodies. Protein sizes are labeled in kDa on the left. Target protein bands are indicated by red asterisks. Protein loading is shown by Ponceau S staining (PS). The predicted molecular weights of GFP‐StKH17, StKNOX3‐GFP, Myc‐StHUB1, and StHUB2 are 59, 73, 103, and 107 kDa, respectively. (C) Representative LUC images of the interaction between StKNOX3 and StHUB1 or StHUB2. (D) Colocalization of StKNOX3‐RFP with GFP‐StHUB1 or GFP‐StHUB2 in transiently infiltrated *N. benthamiana* leaves. Scale bars, 20 μm. (E) BiFC assay showing StKNOX3 interacts with StHUB1 or StHUB2 in the nucleus. Scale bars, 5 μm. (F) Schematic diagram of StKNOX3 showing the domain‐truncated mutants. (G) The KNOX II domain (KD II) of StKNOX3 is required for interaction with StHUB1 in yeast. The Y2H assay was performed as described in (A). (H) StKNOX3^ΔKD II^ mutant loses its interaction with StHUB1, StHUB2, and Pi22798 *in vivo*. Co‐IP assays were performed as described in (C). Protein sizes are labeled in kDa on the left. Target protein bands are indicated by red asterisks. The predicted molecular weights of EV‐GFP, GFP‐StHUB1, GFP‐StHUB2, GFP‐Pi22798, StKNOX3‐RFP, and StKNOX3^ΔKD II^‐RFP are 27, 128, 132, 45, 80, and 66 kDa, respectively.

Normally, KNOX TFs form complexes with other TF families or with transcriptional regulators to modulate downstream gene expression (Kim et al., 2013). Through yeast two‐hybrid (Y2H) screening, StHUB1 (E3 ubiquitin‐protein ligase BRE1‐like 1, XM_006366868.2), an E3 ligase responsible for histone H2B monoubiquitination (forming H2Bub1), was identified as a potential interactor of StKNOX3 (Data S1). The interaction between StKNOX3 and StHUB1 was confirmed by Y2H pairwise assays (Figure 1A). There are two homologs (HUB1 and HUB2) in *Arabidopsis* and in potato (Figure S1) (Cao & Ma, 2011). We then cloned the full‐length StHUB1 and StHUB2 and performed a Co‐IP assay with StKNOX3 in agro‐infiltrated *Nicotiana benthamiana* leaves. The nuclear‐localized potato RNA binding protein StKH17 was included as a non‐interacting control (McLellan et al., 2020). Co‐IP results showed that KNOX3, not control KH17, could be immunoprecipitated by HUB1 and HUB2 (Figure 1B). A split luciferase complementation assay (split‐LCA) further confirmed that StKNOX3 specifically interacts with StHUB1 and StHUB2 (Figure 1C; Figure S2).

Subcellular localization results showed that both StHUB1 and StHUB2 fused with a green fluorescent protein (GFP) at the N terminus localized to the nucleus and cytoplasm when transiently expressed in *N. benthamiana* leaves (Figure S3A–D). When GFP‐StHUB1 or GFP‐StHUB2 was transiently co‐expressed with StKNOX3 in *N. benthamiana*, the fusion proteins co‐localized in the nucleoplasm (Figure 1D; Figure S3C,D). A bimolecular fluorescence complementation (BiFC) assay verified the interactions between StKNOX3 and StHUB1/2 in the nucleoplasm (Figure 1E; Figure S4). These results indicate that StKNOX3 interacts with the H2B monoubiquitination complex composed of StHUB1/2 in the nucleoplasm.

KNOX family members contain four conserved domains, each with distinct functions (Nagasaki et al., 2001). To determine which domain is essential for the interaction between StKNOX3 and StHUB1/2, domain‐truncated mutants were generated (Figure 1F). Y2H and Co‐IP assays showed that a KNOX II domain (KD II) deletion mutant, StKNOX3^ΔKD II^, does not interact with StHUB1 or StHUB2 (Figure 1G,H), suggesting that the KD II of StKNOX3 is required for the interaction with StHUB1/2.

### StHUB1 and StHUB2 were stabilized by StKNOX3 in the nucleus

**Figure 2:**
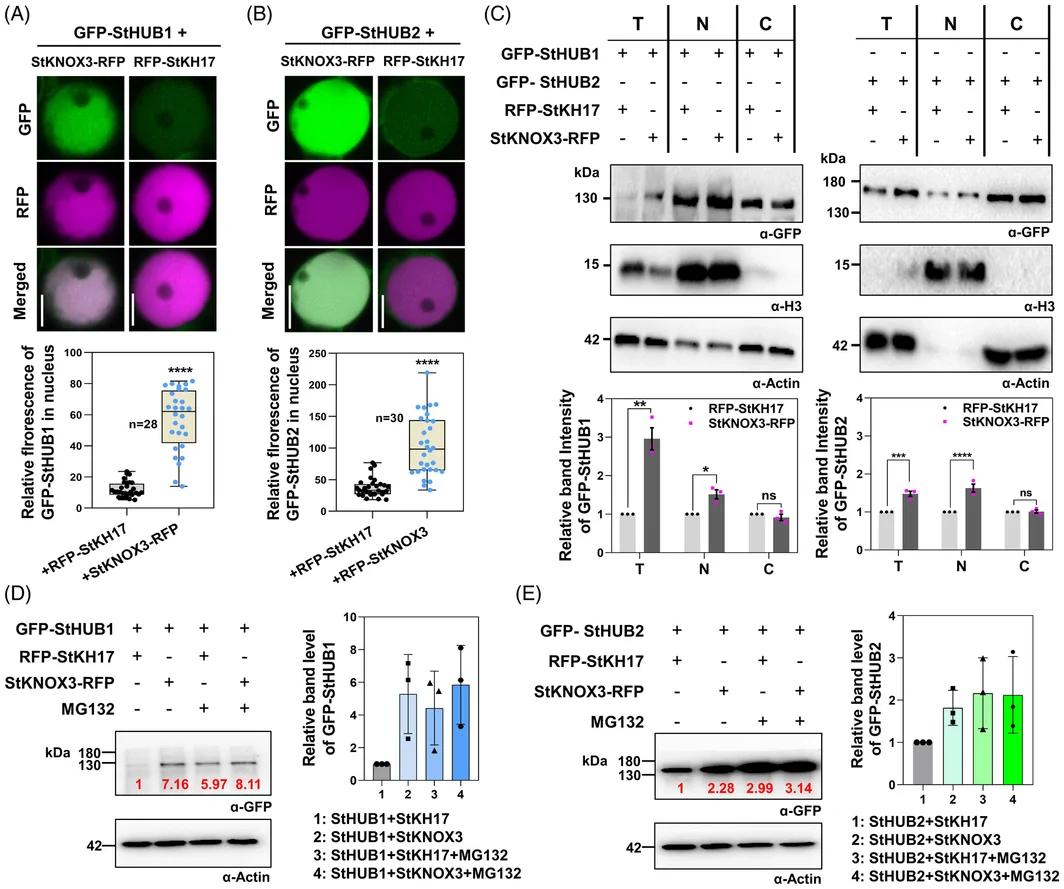
StKNOX3 stabilizes StHUB1 and StHUB2 in the nucleus. (A, B) Fluorescence intensity of GFP‐StHUB1 and GFP‐StHUB2 in the nucleus in the presence of StKNOX3‐RFP or the negative control RFP‐StKH17. Scale bars, 5 μm. Quantification of GFP fluorescence intensity in nuclei is shown in the box graphs beneath the images. Data were analyzed using Student's *t*‐test (*n* = 30, ****P < 0.0001). (C) Cell fraction isolation assays show that protein levels of GFP‐StHUB1 and GFP‐StHUB2 in the nucleus fraction increased when co‐expressed with StKNOX3‐RFP in *Nicotiana benthamiana* leaves, compared to the negative control RFP‐StKH17. C, cytoplasmic protein; N, nuclear protein; T, total protein. To quantify each fraction of GFP‐StHUB1 or GFP‐StHUB2, the GFP band intensities were determined by ImageJ and analyzed by GraphPad. Assays were repeated three times with similar results. Data are mean ± SEM, with asterisks indicating significant differences based on unpaired *t*‐test, *P < 0.05, **P < 0.01, ***P < 0.001, ****P < 0.0001, ns, no significance. Protein sizes are labeled in kDa on the left. The predicted molecular weights of GFP‐StHUB1, GFP‐StHUB2, actin, and histone 3 (H3) are 128, 132, 42, and 15 kDa, respectively. (D, E) The degradation of GFP‐StHUB1 (D) and GFP‐StHUB2 (E) is inhibited by MG132. Actin protein was used as the internal control. For quantification of the total protein level of GFP‐StHUB1 and GFP‐StHUB2, each band was normalized against the actin band from three repeats. Band intensities were determined by ImageJ and analyzed by GraphPad.

Colocalization and BiFC results showed that the interaction between StKNOX3 and either StHUB1 or StHUB2 occurred in the nucleoplasm (Figure 1D,E). We noticed that the fluorescence intensity of GFP‐StHUB1 and GFP‐StHUB2 in the nucleus was significantly increased in the presence of StKNOX3‐RFP compared with that in the presence of RFP‐StKH17 (Figure S3C,D). The GFP fluorescence intensity of GFP‐StHUB1 and GFP‐StHUB2 was quantified when each was co‐expressed with StKNOX3‐RFP or RFP‐StKH17 in *N. benthamiana* cells. Results showed that GFP fluorescence in nuclei was significantly increased in the presence of StKNOX3‐RFP compared with the RFP‐StKH17 control (Figure 2A,B), indicating that StKNOX3 increases the stability of StHUB1 and StHUB2 in the nucleus. The corresponding protein expression levels in total extracts and separated cytoplasmic and nuclear fractions confirmed that nuclear accumulation of StHUB1 and StHUB2 was significantly increased in the presence of StKNOX3‐RFP compared with the presence of RFP‐StKH17 control (Figure 2C).

To explore whether the accumulation of GFP‐StHUB1 or GFP‐StHUB2 in the nucleus is modulated by the 26S proteasome, *N. benthamiana* leaves infiltrated with StKNOX3‐RFP or RFP‐StKH17 and co‐expressed with GFP‐StHUB1 or GFP‐StHUB2 were treated with proteasome inhibitor MG132. The stability of GFP‐StHUB1 and GFP‐StHUB2 was restored by MG132 treatment (Figure 2D,E), indicating that StHUB1 and StHUB2 are turned over by the 26S proteasome *in vivo*. Collectively, these results indicate that StKNOX3 stabilizes StHUB1 and StHUB2 in the nucleus by protecting them from proteolytic degradation mediated by the 26S proteasome.

### StHUB1 and StHUB2 form dimers to regulate H2Bub1 levels and *P. infestans* resistance

**Figure 3:**
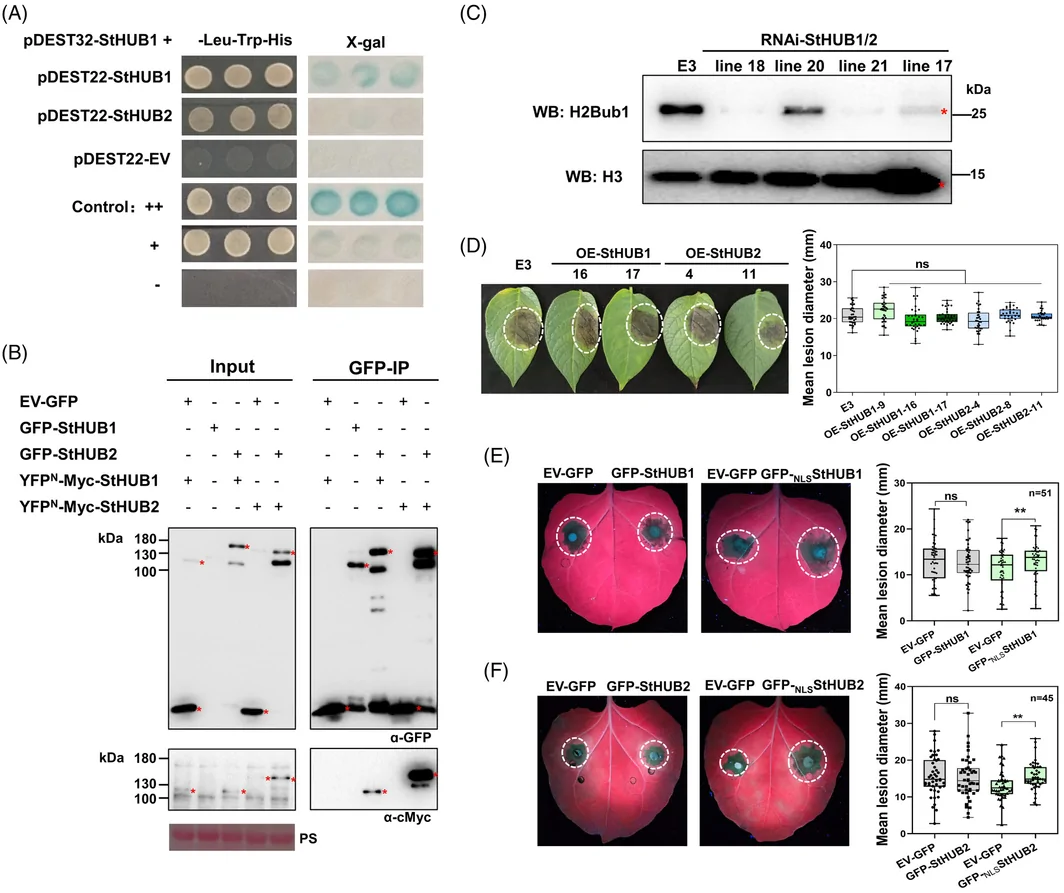
StHUB1 and StHUB2 form dimers to regulate plant H2Bub1 levels and *Phytophthora infestans* resistance. (A) Y2H assay showing the interaction between StHUB1 and StHUB2 or with itself. Yeast co‐expressing pDest32‐StHUB1 with pDest22‐StHUB1 or pDest22‐StHUB2 grew on selective medium SD/‐Trp/Leu/‐His and yielded β‐galactosidase (X‐Gal) activity, but did not when co‐expressed with the empty control. (B) Co‐IP assay showing that StHUB2 interacts with StHUB1 and itself *in vivo*. Total protein was extracted from infiltrated *Nicotiana benthamiana* leaves and IP was performed with GFP beads followed by immunoblotting with anti‐GFP or anti‐cMyc antibodies. Protein sizes are labeled in kDa on the left. Target protein bands are indicated by red asterisks. Protein loading is shown by Ponceau S staining (PS). The predicted molecular weights of EV‐GFP, GFP‐StHUB1, GFP‐StHUB2, YN‐Myc‐StHUB1, and YN‐Myc‐StHUB2 are 27, 128, 132, 125, and 135 kDa, respectively. (C) Silencing both StHUB1 and StHUB2 in potato led to a decreased H2Bub1 level. The nuclear fraction was separated from wild‐type or transgenic potato leaves. Protein sizes are labeled in kDa on the right. The predicted molecular weights of H2Bub1 and H3 are 25 and 15 kDa, respectively. (D) Overexpression of StHUB1 or StHUB2 in potato did not alter *P. infestans* resistance. Results were analyzed with three biological replicates (one‐way ANOVA; ns, no significant difference). (E) Representative leaf images showing the *P. infestans* infection on *N. benthamiana* leaves infiltrated with GFP‐StHUB1, GFP‐_NLS_StHUB1, or empty GFP (EV‐GFP), respectively. The graph shows that overexpression of GFP‐_NLS_StHUB1 in *N. benthamiana* enhances infection of *P. infestans* compared with EV‐GFP (Student's *t*‐test, **P < 0.01, three biological replicates). (F) Representative leaf images showing the *P. infestans* infection on *N. benthamiana* leaves infiltrated with GFP‐StHUB2, GFP‐_NLS_StHUB2, or empty GFP (EV‐GFP), respectively. The graph shows that overexpression of GFP‐_NLS_StHUB2 in *N. benthamiana* enhances infection of *P. infestans* compared with EV‐GFP (Student's *t*‐test, **P < 0.01, three biological replicates).

The fundamental function of HUB1 and HUB2 is to form a heterodimeric complex to facilitate the K120 monoubiquitination of histone H2B (H2Bub1) and thus regulate gene transcription (Cao et al., 2015). Both Y2H pairwise assays and Co‐IP assays verified the interaction between StHUB1 and StHUB2 (Figure 3A,B). To investigate the potential role of HUB1 and HUB2 in plant immunity against *P. infestans*, the expression pattern of *StHUB1* and *StHUB2* was tested during *P. infestans* infection. qRT‐PCR results showed that both *StHUB1* and *StHUB2* transcript abundance was decreased during *P. infestans* infection (Figure S5A,B). We generated transgenic potato plants to either overexpress *StHUB1* (OE‐StHUB1) or *StHUB2* (OE‐StHUB2), or to knock down both *StHUB1* and *StHUB2* (RNAi‐StHUB1/2) simultaneously (Figure S5C,D). The total H2Bub1 level of RNAi plants was decreased compared with wild type (WT) potato variety “E3” plants, indicating a conserved function of StHUB1/2 in regulating H2Bub1 (Figure 3C). These transgenic potato plants were challenged with *P. infestans* strain HB14‐2. Results showed that overexpression of *StHUB1* or *StHUB2* in potato does not alter late blight resistance (Figure 3D). However, simultaneously silencing both *StHUB1/2* genes in potato increased the susceptibility to *P. infestans* (Figure S5E). A potential explanation for this inconsistent resistance is the dual cytoplasm and nuclear subcellular localization of StHUB1 and StHUB2, where they play opposing roles in regulating defense against *P. infestans*.

Our previous work demonstrated that StKNOX3 reduces plant resistance against *P. infestans* (Zhou et al., 2022). In this study, we show that StKNOX3 stabilizes StHUB1 and StHUB2 in the nucleus (Figure 2). We investigated the roles of StHUB1 and StHUB2 in late blight resistance when they were focused in the nucleus. A nuclear localization sequence (NLS, SV40) was fused to the N terminus of StHUB1 and StHUB2 to construct GFP‐_NLS_StHUB1 and GFP‐_NLS_StHUB2, which aimed to localize them exclusively in the nucleus. These constructs were transiently expressed in *N. benthamiana* leaves, and the NLS sequence was able to focus them to the nucleus (Figure S6A,B). EV‐GFP control was expressed in one half of *N. benthamiana* leaves, and either GFP‐StHUB1 or GFP‐_NLS_StHUB1 was transiently expressed in the other half of the leaves. Both sides of the leaves were inoculated with *P. infestans*. Compared with leaves expressing the control GFP, native StHUB1, which localized mainly to the cytoplasm with only weak presence in the nucleus, did not affect susceptibility to *P. infestans* infection (Figure 3E; Figure S6C). By contrast, StHUB1 significantly reduced late blight resistance when localized exclusively in the nucleus in *N. benthamiana* leaves (Figure 3E). Similar results were also observed for StHUB2 (Figure 3F; Figure S6C). Collectively, the above results indicate that StHUB1 and StHUB2 are negative regulators in plant resistance to *P. infestans* when they are in the nucleus.

### Pi22798 promotes the association of StKNOX3 and StHUB1/2

The RXLR effector Pi22798 predominantly localizes in the nucleoplasm and nucleolus, with a cytoplasmic background (Wang et al., 2019; Zhou et al., 2022). Pi22798 contains a nuclear localization signal (NLS) at its C terminus (Figure S7A). An NLS point mutant of Pi22798 (Pi22798_mNLS_) compromises its nuclear localization, as well as its ability to enhance pathogen colonization when expressed in host leaves (Figure S7B,C). A Co‐IP assay showed that Pi22798_mNLS_ does not interact with StKNOX3, confirming that the Pi22798–StKNOX3 interaction occurs in the nucleus (Figure S7D) (Zhou et al., 2022).

**Figure 4:**
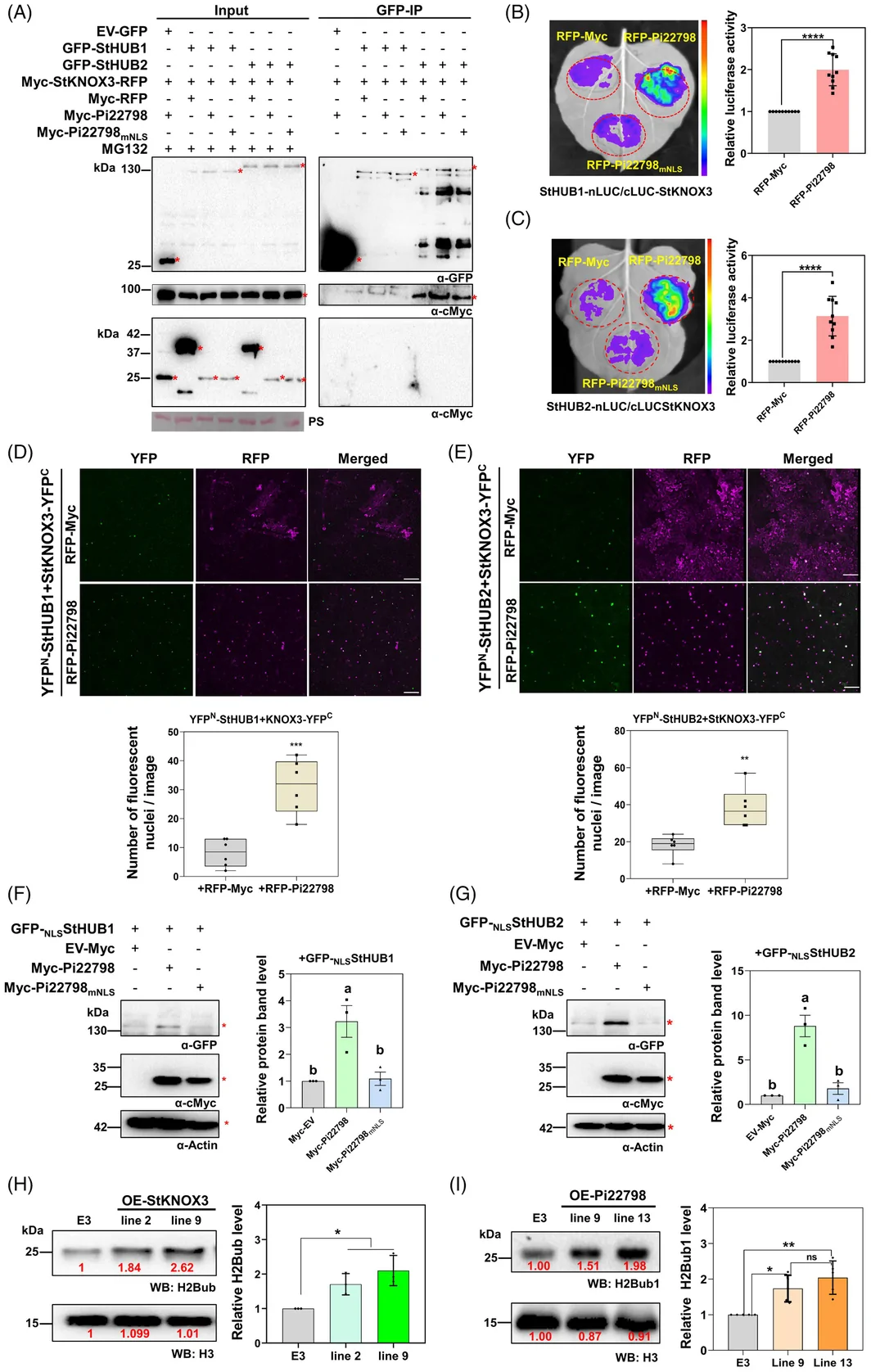
Pi22798 promotes interactions of StKNOX3 with StHUB1 and StHUB2 in the nucleus. (A) Pi22798 promotes the StKNOX3‐StHUB1/2 interactions in a co‐IP assay. Total protein was extracted from infiltrated *Nicotiana benthamiana* leaves and IP was performed with GFP beads followed by immunoblotting with anti‐GFP or anti‐cMyc antibodies. The red asterisks indicate target protein bands of corresponding proteins. Protein sizes are labeled in kDa on the left. The predicted molecular weights of EV‐GFP, GFP‐StHUB1, GFP‐StHUB2, Myc‐StKNOX3‐RFP, Myc‐RFP, Myc‐Pi22798, and Myc‐Pi22798_mNLS_ are 27, 128, 132, 80, 37, 25, and 25 kDa, respectively. Protein loading is shown by Ponceau S staining (PS). (B, C) Split‐LCA assays confirmed that Pi22798 promotes the StKNOX3‐StHUB1 interaction (B) and StKNOX3–StHUB2 interaction (C). *N. benthamiana* leaves were infiltrated with *Agrobacterium* cultures carrying the indicated constructs. Images on the left in (B) and (C) were captured at 48 h after infiltration (hpi) using a chemiluminescent imaging system. The circled quadrants show different combinations of expressed constructs. Graphs on the right show the LUC activity statistics from three replicates of the interactions between cLUC–StKNOX3/StHUB1–nLUC (B) and cLUC–StKNOX3/StHUB2–nLUC (C) (Student *t*‐test, **** *P* < 0.0001). LUC activity was quantified at 48 hpi. (D, E) Representative confocal images show the complementary YFP signals of YFP^N^‐StHUB1/StKNOX3‐YFP^C^ and YFP^N^‐StHUB2/StKNOX3‐YFP^C^ pairs in the nuclei upon transiently co‐expressing with RFP‐Pi22798 or RFP‐Myc. Scare bars, 100 μm. Quantification of the intensity of complementary YFP signals of YFP^N^‐StHUB1/StKNOX3‐YFP^C^ pair in the presence of RFP‐Pi22798 or the control RFP‐Myc (Student *t*‐test; **P < 0.01, ***P < 0.001). Images were taken 2 days post infiltration. Six individual Z‐stacks of 30 slices from three leaves were obtained and analyzed. The fluorescent intensity was measured using Image J software. The data are represented as a Box–Whisker plot. (F, G) Pi22798 promotes the protein stability of StHUB1 and StHUB2 in the nucleus. *N. benthamiana* leaves were infiltrated with *Agrobacterium* cultures carrying the indicated constructs. Total protein was extracted and immunoblotted with anti‐GFP, anti‐cMyc, or anti‐Actin antibodies, respectively. Actin protein was used as the internal control. Protein sizes are labeled in kDa on the left. The red asterisks indicate the target protein bands. The predicted molecular weights of GFP‐_NLS_StHUB1, GFP‐_NLS_StHUB2, Myc‐Pi22798, and Myc‐Pi22798_mNLS_ are 130, 134, 25, and 25 kDa, respectively. For quantification of the total protein level of GFP‐_NLS_StHUB1 and GFP‐_NLS_StHUB2, each GFP band was normalized against the actin band. Band intensities were determined by ImageJ and analyzed by GraphPad. Assays were repeated three times with similar results. Data are mean ± SEM, with different letters indicate significant differences (*P* < 0.01, one‐way ANOVA). (H) Stable overexpression of StKNOX3‐RFP in potato led to increased H2Bub1 levels. The nuclear fraction was separated from wild‐type potato leaves, or leaves expressing StKNOX3‐RFP. Protein sizes are labeled in kDa on the left. The predicted molecular weights of H2Bub1 and H3 are 25 and 15 kDa, respectively. The H2Bub1 band intensities were determined by ImageJ and analyzed by GraphPad. *n* = 3 biological replicates. Data are mean ± SEM, with asterisks indicating significant differences based on unpaired *t*‐test (*P < 0.05). (I) Stable overexpression of HA‐Pi22798 in potato led to increased H2Bub1 level. The nuclear fraction was separated from wild‐type potato leaves or leaves expressing HA‐Pi22798. Protein sizes are labeled in kDa on the left. The H2Bub1 band intensities were determined by ImageJ and analyzed by GraphPad. *n* = 5 biological replicates. Data are mean ± SEM, with asterisks indicating significant differences based on unpaired *t*‐test (*P < 0.05, **P < 0.01, ns, no significance).

Since the KD II domain of StKNOX3 is required for its interaction with both Pi22798 and the StHUB1/2 complex in the nucleoplasm, we hypothesized that Pi22798 may affect the formation of the StKNOX3‐StHUB1/2 complex. To test this hypothesis, we conducted a Co‐IP assay of GFP‐StHUB1 or GFP‐StHUB2 with Myc‐StKNOX3‐RFP in the presence of Myc‐Pi22798 or its loss‐of‐function mutant Pi22798_mNLS_ upon MG132 treatment. The Co‐IP assay showed that GFP‐StHUB1 or GFP‐StHUB2 co‐immunoprecipitated more Myc‐StKNOX3‐RFP in the presence of Myc‐Pi22798 compared to the Myc‐RFP control or the Myc‐Pi22798_mNLS_ controls (Figure 4A). We noticed that Myc‐Pi22798 was not immunoprecipitated by StHUB1 or StHUB2 (Figure 4A). Split‐LCA assays further confirmed that RFP‐Pi22798 enhanced the interactions between StHUB1/2 and StKNOX3, compared with the RFP‐Myc control or the RFP‐Pi22798_mNLS_, as reflected by significantly increased luciferase activity (Figure 4B,C; Figure S7E).

Given that StKNOX3 interacts with Pi22798 and StHUB1/2 complex both in the nucleus, we wondered whether Pi22798 promotes formation of the StKNOX3‐StHUB1/2 complex in the nucleus. We performed BiFC assay of YFP^N^‐StHUB1/StKNOX3‐YFP^C^ pair or StHUB2/StKNOX3‐YFP^C^ pair in the presence of RFP‐Pi22798 or the control RFP‐Myc. Remarkably, RFP‐Pi22798 enhanced the interaction between StKNOX3 and StHUB1 and StHUB2 in the nucleus, as reflected by significantly increased numbers of nuclei with YFP signals in plant cells (Figure 4D,E). Potentially as a result of promoting StKNOX3‐StHUB1/2 interactions, we observed that the protein stability of StHUB1 and StHUB2 in the nucleus was clearly enhanced when these proteins were transiently co‐expressed with Pi22798 in *N. benthamiana* leaves (Figure 4F,G). Collectively, these results indicate that Pi22798 facilitates the formation of the StKNOX3‐StHUB1/2 complex in the nucleus.

### StKNOX3 and Pi22798 increase the monoubiquitination of potato H2B

Association of StHUB1 and StHUB2 modulates histone H2B monoubiquitination (H2Bub1), leading to changes in gene transcription (Zarreen et al., 2022). Given that StKNOX3 enhances the stability of StHUB1 and StHUB2 in the nucleus, we hypothesized that overexpression of StKNOX3 may enhance the levels of H2Bub1 in potato. To test this hypothesis, we investigated the H2Bub1 levels in StKNOX3‐OE transgenic plants, compared to untransformed WT “E3” potato. Nuclear fractions from potato leaves were isolated, and the H2Bub1 levels were detected. As shown in Figure 4(H), increased levels of H2Bub1 were observed in potato leaves overexpressing StKNOX3‐RFP. Given that Pi22798 promotes the formation of StKNOX3‐StHUB1/2 complex, it is hypothesized that H2Bub1 levels should be increased also in the Pi22798‐OE plants. We thus compared H2Bub1 levels in the Pi22798‐OE plants and WT “E3” plants. As expected, increased levels of H2Bub1 were also observed in transgenic potato leaves overexpressing HA‐Pi22798 (Figure 4I). Collectively, these results indicate that Pi22798 interacts with StKNOX3 to stabilize StHUB1 and StHUB2 in the nucleus, thus promoting increased H2Bub1 levels.

### StKNOX3 targets specific motifs in the promoter regions of various genes

**Figure 5:**
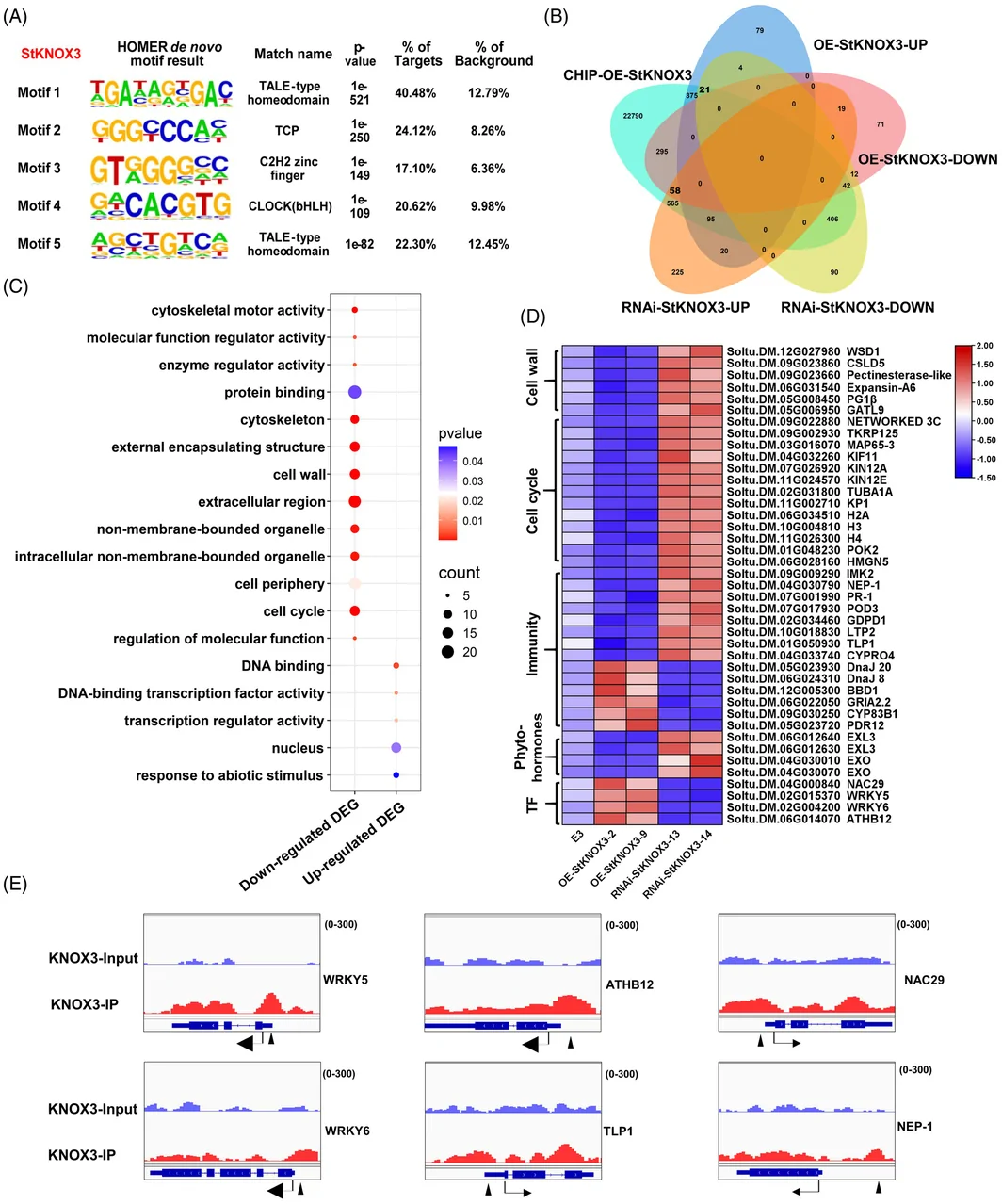
The characteristics of StKNOX3‐binding sites and target genes. (A) HOMER *de novo* motif enrichment analyses of StKNOX3‐binding peaks. The top five significantly enriched binding motifs and their corresponding TF families were presented. (B) Venn diagram showing the number of distinct and overlapping genes between the StKNOX3‐binding targets revealed by ChIP‐Seq and the DEGs identified by RNA‐Seq in StKNOX3‐overexpressing and knockdown lines. (C) Heat map displaying the *P*‐values of GO term enrichment analysis for 79 genes common to DEGs and ChIP‐Seq. (D) Heat map representation of the expression of StKNOX3‐bound DEGs potentially involved in stress responses. (E) StKNOX3‐binding profiles for transcription factor and immunity‐associated genes were visualized with the IGV. Arrows in the gene model indicate transcription start sites. Triangles indicate binding peaks identified by ChIP‐Seq.

To understand the mechanism underlying the functions of StKNOX3 in plant immunity, we performed chromatin immunoprecipitation sequencing (ChIP‐Seq) analysis using the *35S::*StKNOX3‐RFP transgenic line StKNOX3‐OE to identify the binding sites of StKNOX3 (Figure S8). Six‐week‐old potato plants were sampled for library preparation and sequencing. In the StKNOX3‐OE sample, over 6.5 and 5.5 million clean reads were produced from input and immunoprecipitation (IP) libraries, respectively. Approximately 4.4 million (79.40%) reads were mapped to unique positions in the potato DM genome (Genome assembly DM version 6.01), with most of the reads distributed in intergenic regions (Figure S9A). The model‐based analysis of ChIP‐Seq (MACS) peak calling tool was used to identify StKNOX3 binding sites. In total, 57 508 peaks were identified on all chromosomes in the StKNOX3‐OE sample (Data S2). The genome‐wide distribution of StKNOX3 binding sites revealed that about 30% of peaks were located in the promoter regions of genes (Figure S9B,C). Detailed read distribution and peak profile analyses revealed that StKNOX3 was mainly bound to the transcription start site (TSS) regions (Figure S9D). To identify the binding motifs of StKNOX3, we used the HOMER tool to characterize the *de novo* sequence motifs. Results showed that the top five motifs from the HOMER *de novo* motif enrichment analysis were strongly enriched (Figure 5A). Specifically, the binding of StKNOX3 mainly to the core motif containing two copies of the sequence “TGAC” was consistent with previous findings for TALE‐HD proteins (Figure 5A) (Bolduc & Hake, 2009). In addition, another core motif “TGTCA” was also a conserved core sequence recognized by the TALE superclass (Figure 5A) (Nagasaki et al., 2001).

### StKNOX3 modulates gene expression in different pathways

To identify the genes directly regulated by StKNOX3, genes with peaks within 2 kb upstream of the TSS to 1 kb downstream of the transcription end site (TES) region were chosen. We identified 24 252 genes in StKNOX3‐OE (Figure 5B). To further confirm the target genes of StKNOX3, we generated transgenic plants in which *StKNOX3* expression was knocked down in potato, and they exhibit enhanced resistance against *P. infestans* (Figure S10). We collected the wild‐type “E3” potato, two overexpressing and two knockdown transgenic plants of StKNOX3, sampled at the same time as for the ChIP assays, and prepared for RNA‐Seq, respectively. Six hundred upregulated and 497 downregulated genes were identified in *StKNOX3*‐overexpressing plants (Figure 5B; Data S3). Nine hundred and eighty‐two upregulated and 575 downregulated genes were identified in *StKNOX3*‐knock‐down plants (Figure 5B; Data S3). Integrating DEGs (differentially expressed genes) (obtained by RNA‐Seq) and binding target (ChIP‐Seq) data revealed a total of 58 genes that were downregulated in the overexpression lines while upregulated in the knock‐down lines. These genes also overlapped with the target genes identified by ChIP‐Seq (Figure 5B; Data S3). Conversely, a total of 21 genes were upregulated in the overexpression lines while downregulated in the knockdown lines, and these genes coincided with the target genes identified by ChIP‐Seq (Figure 5B; Data S3). Therefore, we consider these DEGs to be the direct target genes of StKNOX3.

To further identify the functions of the StKNOX3 target genes, gene ontology (GO) analysis of the 79 targeted DEGs revealed that GO terms “DNA binding” and “transcription regulator” activity are significantly enriched, indicating that StKNOX3 plays an important role in transcriptional regulation (Figure 5C; Data S4), as anticipated. In addition, GO terms related to “cell wall,” “cytoskeleton,” and “stimulus response” were also enriched in StKNOX3 targeted genes (Figure 5C; Data S4). Among these genes, we noticed a number involved in the cell wall composition, cytoskeleton activity, plant immunity, phytohormone responses, and transcription regulators (Figure 5D). We identified genes encoding TF orthologs in *Arabidopsis* or rice, *WRKY5*, *WRKY6*, *ATHB12*, and *NAC29*, which are reported to play important roles in plant immunity (Choi et al., 2015; Lu et al., 2024; Park et al., 2011; Zhang et al., 2023). Many DEGs identified in the StKNOX3‐OE sample contain obvious StKNOX3 binding peaks in their promoter regions (Figure 5E). In addition, thaumatin‐like protein 1 (LTP1, belonging to the PR5 family of pathogenesis‐related proteins) (Cui et al., 2021; Jin et al., 2001), and aspartic proteinase nepenthesin‐1‐like (NEP‐1, belonging to the group of antifungal proteins AFPs) (Guevara et al., 2001), which function in immunity, both displayed direct binding and transcriptional repression by StKNOX3 (Figure 5E). Taken together, these data suggest that StKNOX3 affects transcriptome reprogramming that participates in cell wall composition, phytohormone responses, and stress responses, including the suppression of many defense‐related genes.

### StKNOX3 directly binds to the promoters of certain defense‐related genes

**Figure 6:**
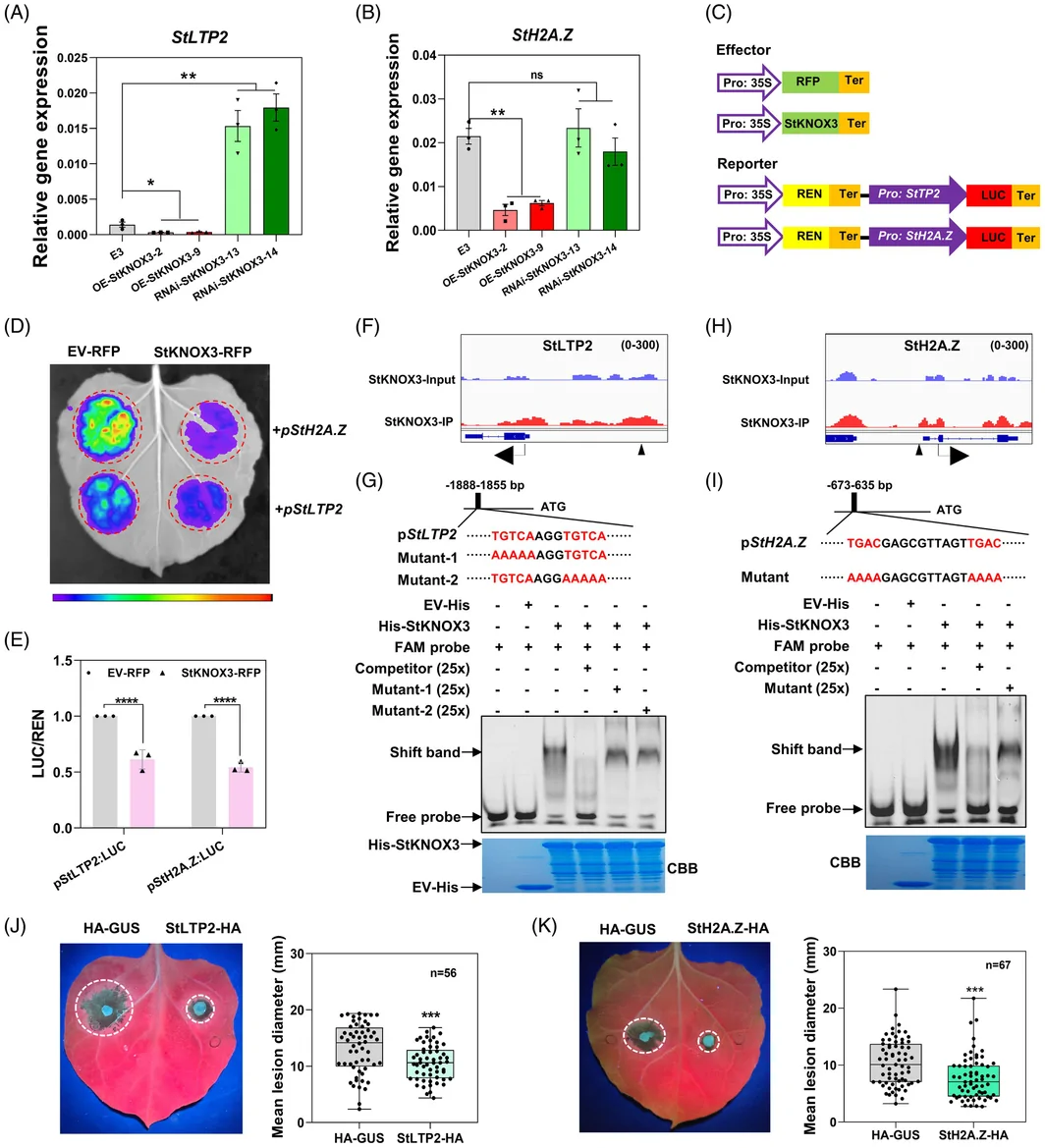
StKNOX3 binds to the promoters of late blight resistance genes and represses their expression. (A, B) qRT‐PCR analysis of *StLTP2* and *StH2A.Z* expression in leaves of wild‐type (E3), *StKNOX3*‐OE and RNAi‐*StKNOX3* potato lines. Expression levels are relative to *Tubulin* (*Tub*). Data are means ± SEM of three replicates. Student's *t*‐test versus “E3”: *P < 0.05, **P < 0.01, ns, not significant. (C) Schematic diagrams of the effector and reporter constructs used in dual luciferase assays. (D) Representative bioluminescence image of *Nicotiana benthamiana* leaves injected with the indicated vector combinations. (E) Relative LUC/REN ratios in *N. benthamiana* leaves expressing effectors (RFP or StKNOX3) with reporters (pStLTP2 or pStH2A.Z). The LUC/REN ratio of the negative control (EV‐RFP) was normalized to 1. Data are mean ± SEM, with asterisks indicating significant differences based on Student *t*‐test (**** *P* < 0.0001). (F, H) IGV visualizations of StKNOX3‐binding profiles at the *StLTP2* and *StH2A.Z* loci. (G, I) EMSA analysis showing binding of StKNOX3 to TGTCA or TGAC *cis*‐acting elements. The indicated 5‐(and‐6)‐Carboxyfluorescein (FAM) labeled probes were incubated with or without recombinant StKNOX3 proteins. Coomassie Brilliant Blue (CBB) staining shows protein loading (bottom panels). (J, K) Functional roles of StLTP2 and StH2A.Z in regulating late blight resistance. Representative images of *Phytophthora infestans*‐infected *N. benthamiana* leaves overexpressing *StLTP2‐HA*, *StH2A.Z‐HA*, or *HA‐GUS*. Graphs showing that overexpression of *StLTP2‐HA* and *StH2A.Z‐HA* significantly decreases infection area compared to *HA‐GUS*. Data are means ± SEM from three biological replicates (Student's *t*‐test, ***P < 0.001).

To investigate whether StKNOX3 directly binds to and negatively regulates defense‐related gene expression, two potential target genes (*StLTP2* and *StH2A.Z*) were selected (Berriri et al., 2016; Chen et al., 2024; Zhao et al., 2025). Their promoters contain either two TGAC motifs or the TGTCA motif—sequences potentially recognized by StKNOX3 (Figure S11). qRT‐PCR analysis confirmed that, consistent with the transcriptome data, both genes were downregulated in *StKNOX3*‐overexpressing lines (Figure 6A,B). The promoter regions of two genes containing the potential StKNOX3‐binding motif were fused with the luciferase gene to generate reporter constructs p*StH2A.Z::LUC* and p*StLTP2::LUC*, respectively (Figure 6C). When StKNOX3 was co‐expressed with these reporter constructs in *N. benthamiana* leaves, weaker luciferase signals were detected compared to when EV‐RFP was co‐expressed with the reporter constructs (Figure 6D,E), demonstrating that StKNOX3 represses the expression of *StLTP2* and *StH2A.Z*.

To test whether StKNOX3 directly binds the promoters of these genes, an electrophoretic mobility shift assay (EMSA) was employed and binding sites were chosen according to the ChIP‐Seq analysis (Figure 6F,H). Results showed that StKNOX3 directly bound to the TGTGA‐containing region (WT FAM probe) in the *StLTP2* promoter and to the two TGAC‐containing regions in the *StH2A.Z* promoter. Moreover, the band intensity decreased upon the addition of unlabeled competitor DNA probes. As anticipated, the motif‐mutated DNA probes failed to compete with the WT probe for StKNOX3 binding (Figure 6G,I). Collectively, these results demonstrated that StKNOX3 directly binds to and represses the expression of the defense‐related genes by targeting their promoters.

To further determine whether *LTP2* and *H2A.Z* play roles in plant resistance against *P. infestans*, these genes were cloned from potato “E3” variety and transiently expressed in half of a leaf of *N. benthamiana*, with *HA‐GUS* expressed as a control in the other half leaves. Following inoculation with *P. infestans* strain 88 069, results showed that overexpression of these genes reduces late blight susceptibility compared to the control (Figure 6J,K; Figure S12). Together, these data demonstrate that StKNOX3 negatively regulates *P. infestans* resistance by directly repressing defense‐related genes.

### Pi22798 enhances the transcription‐suppressive ability of the StKNOX3‐StHUB1/2 complex

**Figure 7:**
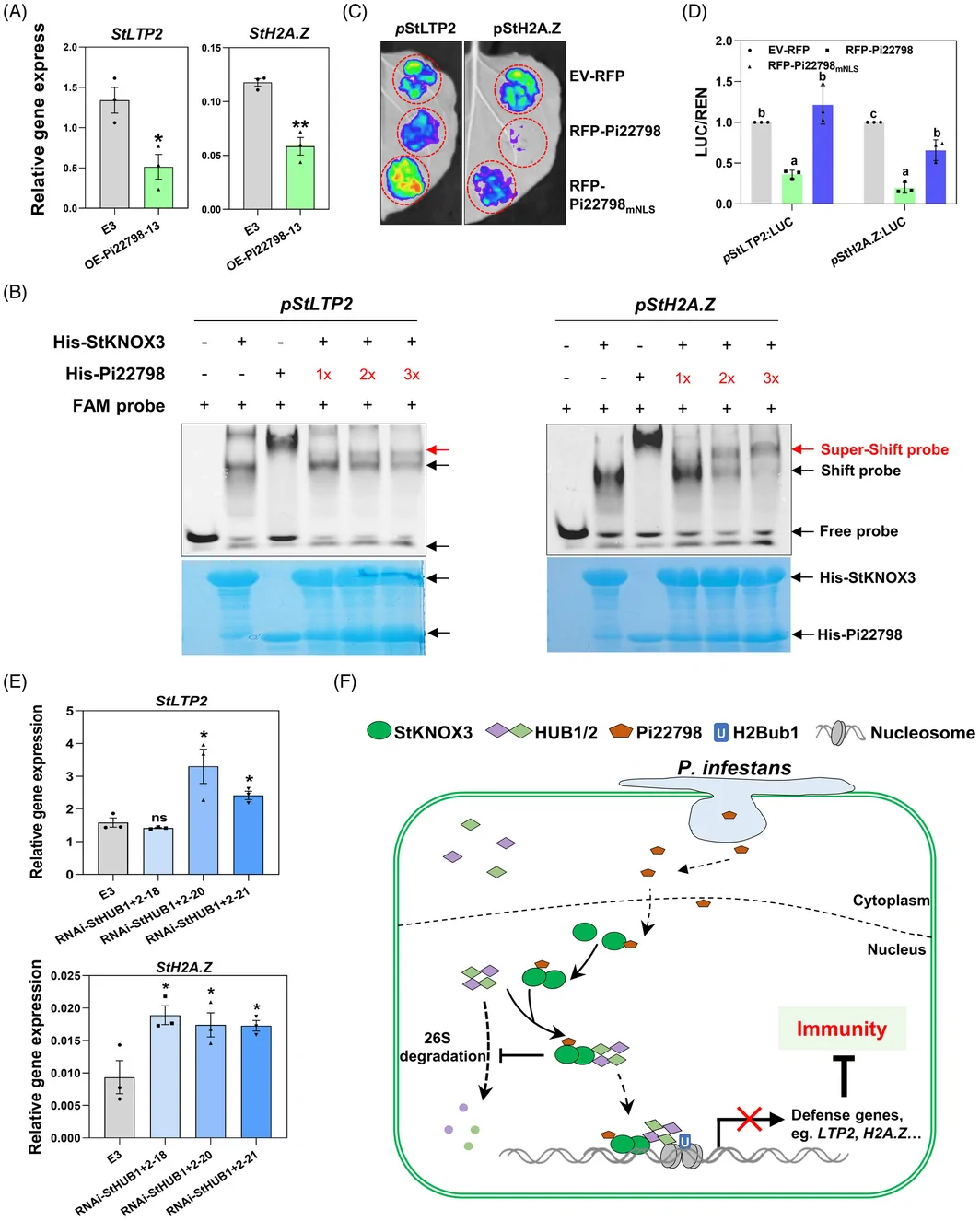
Pi22798 promotes the transcriptional repressor activity of the StKNOX3‐StHUB1/2 complex. (A) qRT‐PCR analysis of *StLTP2* and *StH2A.Z* expression in leaves of wild‐type (E3) and *HA‐Pi22798* overexpression lines. Expression levels were normalized to *Tubulin* (*Tub*). Data are means ± SEM from three biological replicates. Statistical significance relative to “E3” was determined by Student's *t*‐test: *P < 0.05; **P < 0.01. (B) Pi22798 increases the DNA‐binding affinity of StKNOX3 for TGAC or TGTCA elements in promoter regions. FAM‐labeled probes were incubated with or without recombinant proteins as indicated. Protein loading is shown by CBB staining (bottom panel). (C) Representative bioluminescence image of an *Nicotiana benthamiana* leaf co‐infiltrated with the indicated vector combinations. (D) Relative LUC/REN ratios in *N. benthamiana* leaves transiently expressing effectors (RFP, RFP‐Pi22798, or RFP‐Pi22798_mNLS_) along with reporters containing the *StLTP2* or *StH2A.Z* promoters. The LUC/REN ratio for the negative control (EV‐RFP) was set to 1. (E) qRT‐PCR analysis of *StLTP2* and *StH2A.Z* expression in leaves of wild‐type (E3) and dual *StHUB1/2* RNAi lines. Expression levels were normalized to *Tubulin* (*Tub*). Data are means ± SEM from three biological replicates. Statistical significance relative to “E3” was determined by Student's *t*‐test: *P < 0.05; ns, not significant. (F) A working model illustrating how Pi22798 targets the StKNOX3‐StHUB1/2 subcomplex to suppress the host defense. Pi22798 promotes StKNOX3 homodimerization and recruits H2B monoubiquitination regulators StHUB1 and StHUB2 in the nucleus. These interactions enhance the protein stability of StHUB1 and StHUB2, leading to elevated H2Bub1 levels. Consequently, increased H2Bub1 level reinforces StKNOX3‐mediated transcriptional repression of defense‐related genes, thereby compromising plant immunity during *Phytophthora infestans* infection.

Our previous work showed that Pi22798 promotes StKNOX3 homodimerization to enhance susceptibility (Zhou et al., 2022). We thus tested whether Pi22798 influences the DNA‐binding ability of StKNOX3 to the promoter of its targeted genes as well as its suppressive function on defense‐related genes. *StLTP2* and *StH2A.Z* were selected as candidate genes to test this hypothesis. As shown in Figure 7(A), the expression of both genes was decreased in *Pi22798*‐OE potato lines compared to wild‐type “E3” plants.

Electrophoretic mobility shift assays (EMSAs) showed that His‐Pi22798 alone does not bind to the *StLTP2* and *StH2A.Z* promoter probes. However, increased concentration of His‐Pi22798 promoted the formation of a Pi22798–StKNOX3 complex, which enhanced the binding of StKNOX3 to these probes, as evidenced by stronger super‐shifted bands in EMSA (Figure 7B). To investigate whether Pi22798 affects the transcriptional suppressor activity of StKNOX3 on these genes, an LUC assay was performed. Lower luciferase activity was observed in the presence of Pi22798 compared to the EV‐RFP control and the mutant Pi22798_mNLS_ (Figure 7C,D). The above results demonstrate that Pi22798 promotes the binding ability of StKNOX3 to the promoters of defense‐related genes, thereby potentiating its repressive function on their expression.

The association of StHUB1 and StHUB2 modulates the H2Bub1 level and transcriptional regulation. We speculated that StKNOX3 recruits StHUB1 and StHUB2 to increase the H2Bub1 level, thereby synergistically suppressing defense‐related gene expression. In line with that, the expression level of *StLTP2* and *StH2A.Z*, which were downregulated in *StKNOX3*‐OE and *Pi22798*‐OE plants, was upregulated in the dual *StHUB1/2* RNAi plants (Figure 7E). Collectively, our data support a model in which Pi22798 promotes StKNOX3 homodimerization and its association with StHUB1/2 in the nucleus. The increased H2Bub1 level reinforced the ability of StKNOX3 to effectively suppress defense‐related gene expression, which consequently compromises plant immunity (Figure 7F).

## DISCUSSION

Studies have identified KNOX class II transcription factors as key regulators involved in plant growth, development, and immunity responses (Azarakhsh et al., 2015, 2020; Chen et al., 2023, 2025; Di Giacomo et al., 2017; Hackbusch et al., 2005; Kim et al., 2013; Pagnussat et al., 2007; Sheng et al., 2022). In our previous work, we identified the potato StKNOX3 as a host target of *P. infestans* effector Pi22798, which modulates StKNOX3 activity to negatively regulate defense against *P. infestans* (Zhou et al., 2022). Here, we demonstrate that StKNOX3 interacts with StHUB1 and StHUB2, two E3 ubiquitin ligases responsible for monoubiquitinating histone H2B to generate H2Bub1 in the nucleus. Elevated nuclear H2Bub1 reinforces StKNOX3‐mediated transcriptional repression of defense‐related genes, thereby promoting *P. infestans* colonization (Figures 1, 2, 3, 6, and 7). An integrated ChIP‐Seq and RNA‐seq analysis revealed the genome‐wide downstream binding targets of StKNOX3, several of which are implicated in resistance against *P. infestans* (Figures 5 and 6). The effector Pi22798 promotes formation of the StKNOX3‐StHUB1/2 complex, which enhances its transcriptional repressive activity and consequently suppresses host defense gene expression (Figures 4 and 7).

HOS59, a homolog of StKNOX3 in rice, regulates rice grain size and resistance to *Magnaporthe oryzae* and *Xoo* (Sheng et al., 2022; Xu et al., 2024). Its downstream target genes are primarily associated with plant structural morphology and stress traits, suggesting that HOS59 exerts broad functions and may play an important role in the balance between plant growth and stress responses. In this work, we found that StKNOX3 binds to the promoters of genes associated with cell wall biogenesis, plant immunity, and the brassinosteroids (BR) phytohormone pathway (Figure 5), indicating that potato StKNOX3 contributes to the regulation of disease responses. We confirmed that StKNOX3 directly represses the expression of two genes implicated in resistance to *P. infestans* (Figure 6). In addition to its role in defense, StKNOX3 was also found to regulate numerous cell cycle‐related genes (Figure 5C,D). Consistently, its rice homolog has been implicated in cyclin‐mediated cell cycle regulation (Cooper et al., 2003). Our previous work showed that overexpression of StKNOX3 in *N. benthamiana* and potato plants affects growth and morphology, resulting in slower growth rate and thicker, darker green leaves (Zhou et al., 2022). Therefore, further investigation into the effects of StKNOX3 on plant growth and development is warranted.

StKNOX3 is a KNOX II transcription factor (TF). Previous studies have demonstrated that its orthologs in *Arabidopsis*, rice, and lettuce interact with other TF families, such as OFPs, BELLs, and BLHs, to form transcriptional complexes that regulate downstream genes (Chen et al., 2025; Cooper et al., 2003; Schmitz et al., 2015; Sheng et al., 2022). Moreover, HOS59 interacts with some epigenetic regulators, including PIWI domain‐containing proteins and several key enzymes involved in the methionine cycle, suggesting an essential role in DNA methylation and gene silencing (Sheng et al., 2022). In our yeast library screen, no canonical transcription factors were identified as StKNOX3 interactors (Data S1). Surprisingly, we identified an epigenetic modification regulator, StHUB1, as a potential interactor of StKNOX3. HUB1 and HUB2 have been reported to play important roles in regulating defense and immune responses (Zarreen et al., 2022). In *Arabidopsis* and rice, HUB1 and HUB2 localize predominantly to the nucleus (Cao et al., 2015; Liu et al., 2007; Wen & Ao, 2000). Here, we observed that GFP‐tagged StHUB1 and StHUB2 also exhibited strong cytoplasmic localization in addition to nuclear accumulation (Figure S3A). When targeted to the nucleus, both StHUB1 and StHUB2 reduced plant resistance to *P. infestans* (Figure 3E,F). Our results further showed that StKNOX3 stabilizes StHUB1 and StHUB2 in the nucleus, leading to increased H2Bub1 levels in StKNOX3‐OE transgenic potato plants compared with wild‐type “E3” (Figure 4H). In rice, HOS59 promotes the degradation of the NLR immune regulator BRG8, thereby suppressing BRG8‐mediated immunity (Xu et al., 2024). Our findings suggest that StKNOX3 may similarly recruit StHUB1 and StHUB2 to facilitate *P. infestans* infection. As a KNOX TF, StKNOX3 may function as an adaptor that can prevent specific E3 ubiquitin ligases from mediating StHUB1 and StHUB2 degradation. Notably, under normal localization conditions, StHUB1 and StHUB2 exhibited no obvious role in regulating resistance to *P. infestans* (Figure 3D–F). We speculate that nuclear and cytoplasmic pools of StHUB1 and StHUB2 may play distinct roles during infection, and further studies are needed to dissect their compartment‐specific functions.

The modification of histone H2Bub1 is well conserved from yeasts to humans and plants. Silencing both *HUB1* and *HUB2* in potato markedly reduced global H2Bub1 levels (Figure 3C), indicating a conserved role of StHUB1 and StHUB2 in regulating H2Bub1 deposition. Changes in *HUB1* and *HUB2* expression have been reported in response to bacterial, viral, and fungal infections, often leading to altered disease resistance. For instance, transcripts of tomato *SlHUB1* and *SlHUB2* are induced upon infection by *B. cinerea and P. syringae* pv. *tomato* DC3000 (Zhang, Li, et al., 2015; Zhang, Liu, et al., 2015). Similarly, *AtHUB1* is upregulated in *Arabidopsis* following challenge with *Pst* DC3000, *A. brassicicola*, *Erysiphe cichoracearum*, and *Cochliobolus carbonum* (Dhawan et al., 2009; Zou et al., 2014). In contrast, *OsHUB1* and *OsHUB2* are downregulated during compatible interactions between rice and *M. oryzae* (Zhang, Li, et al., 2015; Zhang, Liu, et al., 2015). These expression changes frequently alter H2Bub1 levels at specific disease resistance gene loci—such as *SNC1* and *RbohD*—during pathogen infection, thereby shaping immune outcomes (Zhao et al., 2020; Zou et al., 2014). In our study, we found that the expression of *StHUB1* and *StHUB2* was decreased during *P. infestans* infection (Figure S5A,B). However, how H2Bub1 dynamics are regulated during *P. infestans* infection remains unclear. Given that StKNOX3 stabilizes StHUB1 and StHUB2 in the nucleus and elevates global H2Bub1 levels in potato, it is worthwhile to investigate whether the target genes regulated by StKNOX3 are modified by H2Bub1, and whether this modification changes during pathogen infection.

Our previous work showed that Pi22798 interacts with StKNOX3 in the nucleus and the KNOX II domain (KD II) of StKNOX3 is required for this interaction (Zhou et al., 2022). In this study, colocalization, BiFC, and nuclear‐cytosolic fractionation assay consistently demonstrate that StHUB1/2 also interacts with StKNOX3 within the nucleus (Figures 1 and 2). Moreover, confocal imaging reveals that Pi22798 markedly enhances the nuclear fluorescence intensity of the StKNOX3‐StHUB1/2 interactions (Figure 4D,E). Notably, the KD II domain is also essential for the interaction with StHUB1 and StHUB2 (Figure 1F–H). Given that Pi22798 promotes the StKNOX3 homodimerization, our findings support a model in which Pi22798 enhances the nuclear assembly of the StKNOX3‐StHUB1/2 subcomplex, leading to increased stability of StHUB1/2 and elevated global H2Bub1 levels (Figure 4). However, the precise molecular mechanism underlying Pi22798‐mediated subcomplex assembly remains unknown. Further structural studies of protein complexes containing Pi22798, StKNOX3, and StHUB1/2 will help to elucidate this process.

Pathogens often subvert host immunity by manipulating plant transcriptional programs. Oomycetes employ effectors to target host transcription factors and other transcriptional regulators, thereby repressing defense gene expression or inducing susceptibility (Fabro, 2022). For example, *P. sojae* effector PsAvh52 recruits the host cytoplasmic acetyltransferase GmTAP1 into nuclear speckles to promote epigenetic modifications and enhance plant susceptibility (Li et al., 2018). Another RXLR effector, PsAvh23, disrupts the formation of an ADA2‐GCN5 subcomplex by competitively binding to ADA2, thereby repressing the expression of defense genes by reducing GCN5‐mediated H3K9ac (Kong et al., 2017). The oomycete effector HaRxLL470 enhances pathogenicity by interfering with the bZIP transcription factor AtHY5, attenuating its binding to defense‐related promoters (Chen et al., 2021). Recently, TSWV‐encoded NS proteins were reported to disrupt the dimerization of TCP17 and inhibit its transcriptional activation, leading to the inhibition of the auxin signaling response pathway, thereby promoting viral infection (Zhao et al., 2024). Here, we present evidence for a distinct strategy: effector Pi22798 promotes homodimerization of the susceptibility factor StKNOX3, enhancing its binding affinity to defense‐related gene promoters and repressing their expression (Figure 7) (Zhou et al., 2022). Furthermore, in an EMSA experiment, increasing amounts of Pi22798 weakened the StKNOX3‐probe complex while generating a slower migrating band, suggesting the formation of a larger complex that binds cooperatively to the probe, rather than transient interaction followed by dissociation (Figure 7B). Given that Pi22798 promotes assemblage of the StKNOX3‐StHUB1/2 subcomplex, whether StKNOX3 target genes are also modified by H2Bub1 in *Pi22798‐OE* potato lines requires further investigation to fully understand how Pi22798 represses plant immunity.

Taken together, our study reveals a previously unrecognized strategy of immune suppression: a nuclear‐localized pathogen effector hijacks a host susceptibility transcription factor and couples with HUBs ubiquitin ligases to reprogram plant transcription via epigenetic modifications, ultimately enhancing plant susceptibility.

## MATERIALS AND METHODS

### Plant materials and growth conditions

Wild‐type and transgenic *N. benthamiana* were grown in a growth chamber at 22°C, with a 16‐h light with 8‐h dark photoperiod. Plants at 3–4 weeks old were used for transient expression and *P. infestans* inoculation assays, while 2‐week‐old plants were used for VIGS. For potato plants, wild‐type variety “E‐potato‐3” (E3) and transgenic lines were grown under natural conditions in a greenhouse. Plants at 6–8 weeks old were prepared for *P. infestans* inoculation assays.

### Plasmid constructs

The coding sequence of *StKNOX3*, *StHUB1*, *StHUB2*, and *Pi22798* was amplified by PCR from potato or *P. infestans* cDNA using specific primers, respectively. StKNOX3 and its truncated mutants were cloned into the pHellsgate8 vector with a C‐terminal mCherry tag at *Xho* I and *Eco* RI sites to generate StKNOX3‐RFP and StKNOX3Δ^KD II^, respectively. *Pi22798*, *Pi22798*_*mNLS*_, and *StKH17* were cloned into pK7WGR2 vector at *Bsr* G1 site to form RFP‐Pi22798, RFP‐Pi22798_mNLS_, and RFP‐StKH17 plasmids. *StKNOX3* was cloned into pH7LIC‐C‐GFP vector at *Bam* HI and *Xho* I sites to form StKNOX3‐GFP. *StHUB1*, *StHUB2*, *Pi22798*, and *Pi22798*_*mNLS*_ were recombined into pH7LIC‐N‐GFP vector at *Stu* I site to form GFP‐StHUB1, GFP‐StHUB2, GFP‐Pi22798, and GFP‐ Pi22798_mNLS_. *StHUB1* and *StHUB2* were cloned into pH7LIC‐N‐Myc vector at *Stu* I site to form MYC‐StHUB1, MYC‐StHUB2. *StHUB1* and *StKNOX3* were cloned into entry vector pDONR221 by gateway cloning following the manufacturer's instructions (Invitrogen) and then recombined into yeast expression vectors to generate pDEST32‐StHUB2, pDEST32‐StKNOX3, and pDEST22‐StKNOX3. *StHUB1*, *StHUB2*, *StKNOX3*, and *Pi22798* were cloned into ‐nLUC and ‐cLUC vector between *Kpn* I and *Sal* I sites to form Pi22798‐nLUC, StHUB1‐nLUC, StHUB2‐nLUC, cLUC‐StKNOX3, cLUC‐StHUB1, and cLUC‐StHUB2 plasmids for luciferase complementation assays (LCA). *StHUB1*, *StHUB2*, and *Pi22798* were cloned into pBiFC‐VN173 at the *Bam* H I and *Kpn* I sites to form YFP^N^‐Pi22798, YFP^N^‐StHUB1, and YFP^N^‐StHUB2 plasmids, and *StKNOX3* was cloned into pBiFC‐VC155 at *Xba* I and *Xho* I sites to form StKNOX3‐YFP^C^ plasmids for BiFC detection. For RNAi transgenic lines or transient silencing, specific fragments of *StKNOX3*, *StHUB1*, *StHUB2*, and *NbKNOX3* were inserted into pHELLSGATES8 at *Xho I* and *Xba I* sites. All primers used in this study are listed in Data S5.

### *Agrobacterium*‐mediated transient gene expression assays

All constructs were transformed into *Agrobacterium tumefaciens* strain GV3101. *Agrobacterium* cells were grown in YEB medium supplemented with the appropriate antibiotics at 28°C with shaking overnight. The prepared cultures were centrifuged at 4000 ***g*** for 10 min at room temperature (RT), and the pellets were resuspended in MMA medium (10 mM 2‐(N‐morpholino) ethanesulfonic acid, 10 mM MgCl_2_, and 200 mM acetosyringone) and incubated in the dark for at least 30 min before infiltration. Bacterial suspensions were adjusted to an optical density at OD_600_ as follows: 0.01 for protein localization visualization; 0.1 for BiFC, *P. infestans* inoculation assay, and cell fraction isolation experiments; 0.5 for western blot, co‐immunoprecipitation, and LCA. All bacterial suspensions were mixed with the silencing suppressor p19 at a final OD_600_ of 0.05 prior to infiltration.

### Potato and *N. benthamiana* transformation

For transgenic transformation, plasmids were transformed into *Agrobacterium* strain GV3101. The variety “E3” was used for potato transformation using microtuber discs as explant as previously described (Si et al., 2003). Healthy, fully expanded leaves of *N. benthamiana* were used for transformation by leaf disks as previously described (Qi et al., 2024). The explants were selected on Murashige and Skoog (MS) medium containing 100 mg ml^−1^ kanamycin. Positive lines were confirmed by PCR using a 35S promoter forward primer and a gene‐specific reverse primer. Silencing and overexpression efficiency were verified by quantitative real‐time PCR (qRT‐PCR), with *NbActin* and *StTub* used as reference genes. All primers used in this study are listed in Data S5.

### Library screening and Y2H assay

The pDEST32‐StKNOX3 plasmid was transformed into MAV203 yeast strain as the bait. Yeast library screening was carried out according to the protocol as previously reported (Bos et al., 2010). Individual colonies were selected, and the inserts were amplified by PCR for sequencing to identify interacting proteins. The sequence of each candidate was analyzed by using the NCBI BLAST.

A Y2H assay was used to verify the interaction between StKNOX3 and StHUB1. The plasmids pDEST32‐StHUB1 and pDEST22‐StKNOX3 were co‐transformed into MAV203 yeast according to the manufacturer's instructions (Invitrogen, PQ10001‐01 and PQ10002‐01). The positive clones were selected on medium lacking histidine (SD/‐Leu/‐Trp/‐His) and assessed for β‐galactosidase (X‐gal) activity.

### Western blot, Co‐immunoprecipitation (co‐IP) and immunoblot analysis

*Agrobacterium* strain GV3101 containing the relevant plasmids was infiltrated into *N. benthamiana* leaves. Two to 3 days after infiltration, samples were collected and frozen in liquid nitrogen. For western blot, samples were ground and mixed with 200 μl of GTEN buffer (10% (v/v) glycerol, 25 mM Tris–HCl [pH 7.5], 1 mM EDTA, 150 mM NaCl, supplemented with 10 mM DTT, protease inhibitor cocktail [Roche Life Science], 1 mM phenylmethylsulfonyl fluoride [PMSF], and 0.2% NP‐40). Protein lysates were incubated on ice for 30 min with occasional shaking, followed by centrifugation at 12 000 ***g*** for 10 min at 4°C; this step was repeated twice. The cleared supernatants were transferred into new tubes and boiled with an equal volume of 2 × SDS loading buffer at 95°C for 10 min. For Co‐immunoprecipitation (co‐IP), leaves were ground and mixed with 1 ml of GTEN buffer. After the ice incubation and centrifugation, clear supernatants were added with 20 μl of anti‐GFP beads (AlpaLifeBio), followed by 2 h incubation at 4°C with constant rotation. Bead‐bound proteins were collected and washed with washing buffer five times, then boiled with 50 μl 2 × SDS‐loading buffer at 95°C for 10 min.

For immunoblot analysis, samples were loaded onto 8–15% SDS‐PAGE gels and run for 1.5 h at 120 V. The proteins were transferred to polyvinylidene fluoride (PVDF) membranes, followed by blocking in PBS buffer with 5% (v/v) milk for 1 h before the addition of primary antibodies and incubation overnight. After incubation with secondary antibody, the membranes were visualized using chemiluminescent ECL (Servicebio, Wuhan, China). Primary antibodies: anti‐GFP and anti‐cMyc (Bioyears, Wuhan, China) raised in mouse at a dilution of 1:5000; anti‐RFP (EnoGene, Nanjing, China) raised in mouse at a dilution of 1:2500; anti‐H2Bub1 (CST, #5546) raised in rabbit at a dilution of 1:3000; anti‐H3 (Abways) raised in mouse at a dilution of 1:3000; anti‐H^+^‐ATPase (Agrisera) raised in rabbit at a dilution of 1:2500; anti‐Actin (Abbkine) raised in mouse at a dilution of 1:5000. Secondary antibodies: anti‐mouse (MBL) and anti‐rabbit (Biopm) at a dilution of 1:5000.

### Split LCA

*Agrobacterium* strain GV3101 containing the relevant plasmids was infiltrated into *N. benthamiana* leaves. Two to three days after infiltration, the infiltrated areas were sprayed with 10 μl of 100 mM D‐luciferin and kept in the dark for 15 min. The leaves were detached to observe the luminescence using the NightShade LB 985 *in vivo* plant imaging system with indiGO software.

For measurement of luciferase activity, infiltrated *N. benthamiana* leaves were punched into disks and transferred to a white 96‐well microplate containing 200 μl of ddH_2_O overnight. To ensure experimental accuracy, at least eight replicates were used for the same infiltration site. After removing the ddH_2_O from the microplate, 100 μl of reaction solution containing 1 mM luciferin substrate was added into disks and incubated for 5–10 min. Luminescence scanning was performed using a luminometer with a scanning interval of 0.5 sec for 30 cycles. Bar charts were generated based on the scanned values to show the mean intensity of the luminescence intensity resulting from the luciferase‐mediated substrate decomposition, thereby determining the interaction strength between the target proteins. This experiment was repeated three times.

### Confocal microscopy

Leaf cells transiently expressing vector fusions were imaged 48–72 h post‐infiltration using a Leica TCS‐SPE confocal microscope with water‐dipping objectives. GFP was excited with 488 nm light, and the emissions were detected between 492 and 520 nm. Monomeric RFP was excited at 561 nm, and emission was detected at 600–630 nm. YFP was excited with 514 nm light, and emissions were detected between 520 and 550 nm. Co‐expression experiments were performed on at least three independent occasions. Generally, cells displaying a low level of fluorescence were imaged to minimize overexpression artifacts. Fluorescence intensity was quantified using ImageJ and Leica LCS software packages, as required.

### *P. infestans* inoculation assay

*P. infestans* isolates, including 88 069 (for *N. benthamiana*) and HB09‐14‐2 (for potato), were cultivated at 16°C in the dark on rye agar medium plates for 3–4 weeks before inoculation assay. Sporangia were collected and adjusted to a final concentration of 80 spores μl^−1^ for potato and 150 spores μl^−1^ *N. benthamiana* in ddH_2_O, respectively. The prepared inoculum was incubated at 4°C for 1–4 h before inoculation. Lesion diameters and spore numbers were measured at 4–7 days post‐inoculation (dpi).

### Nuclear cytoplasmic protein fraction

Nuclear and cytoplasmic fractions from *N. benthamiana* leaves were separated as previously described (Yu et al., 2024). Briefly, 0.2 g of transiently expressed *N. benthamiana* leaves were homogenized in 1 ml of pre‐chilled lysis buffer (20 mM Tris‐HCl, pH 7.5, 20 mM glycerol, 250 mM sucrose, 5 m KCl, 2 mM EDTA, 2.5 mM MgCl_2_, 2.5% DTT, and 1 × protease inhibitor cocktail). After filtration through two layers of Miracloth, 50 μl of the flow‐through was transferred to a new 1.5 ml tube and used as the total lysate (T). The rest of the flow‐through was centrifuged at 1500 ***g*** for 10 min at 4°C. The pellets from the 1500 ***g*** spin were resuspended in 8 ml of nuclear resuspension buffer 1 (NRB1: 20 mM Tris‐HCl, pH 7.4, 2.5 mM MgCl_2_, and 0.2% Triton X‐100) by pipetting five to eight times. When the pellet turned white, it was resuspended with 500 μl NRB2 (20 mM Tris‐HCl, pH 7.5, 250 mM sucrose, 0.5% Triton X‐100, 10 mM MgCl_2_, 5 mM beta‐mercaptoethanol, and 1 × protease inhibitor cocktail), then carefully overlaid on top of 500 μl NRB3 (20 mM Tris‐HCl, pH 7.5, 0.5% Triton X‐100, 10 mM MgCl_2_, 1.7 M sucrose, 5 mM beta‐mercaptoethanol, and 1 × protease inhibitor cocktail). The sample was centrifuged at 16 000 ***g*** for 45 min at 4°C, and the final pellet was dissolved in 50 μl of Minute™ non‐denatured protein solubilization reagent (Invent Biotechnologies, WA‐010), as “N” (nuclear) fraction. This fraction was mixed with an equal volume of 2 × SDS loading buffer and boiled at 95°C for 10 min. The supernatant from the 1500 ***g*** spin was centrifuged at 16 000 ***g*** for 10 min. A 400‐μl aliquot of the supernatant was precipitated with 1.6 ml pre‐chilled acetone overnight at −20°C, then centrifuged for 10 min at 16 000 ***g*** and 4°C, to obtain the “C” (cytoplasmic) fraction. This fraction was mixed with 50 μl 2 × SDS‐loading buffer, boiled at 95°C for 10 min and prepared for immunoblotting.

### RNA extraction and qRT‐PCR analysis

Total RNA was extracted from potato or *N. benthamiana* leaves using a Plant Total RNA Kit (ZOMANBIO). First‐strand cDNA was synthesized using a reverse transcriptase kit (Abm). Quantitative PCR was performed on an ABI7300 PCR machine (Applied Biosystems) using the Blastaq 2 × qRT‐PCR Mastermix (Abm) according to the manual. Primers for qRT‐PCR are listed in Data S5, and gene expression levels were analyzed using the 2^−ΔΔCT^ method described by Livak and Schmittgen (2001). Gene expression was normalized to the internal control *StTub* or *NbActin*.

### ChIP seq and data analysis

ChIP experiments and high‐throughput sequencing and data analysis were conducted by Seqhealth Technology Co., Ltd (Wuhan, China). ChIP assay was performed on *35S::StKNOX3‐RFP* transgenic potato lines (StKNOX3‐OE). One gram of leaf tissue was fixed in 1% formaldehyde for 10 min at room temperature under vacuum, after which 0.125 M glycine was added and the mixture was incubated for 5 min to terminate the cross‐linking reaction. The tissue was then collected, frozen in liquid nitrogen, and ground using a tissue lyser. The ground powder was treated with cell lysis buffer, and nuclei were collected by centrifugation at 2000 ***g*** for 5 min. Then, the pellet was treated with nuclear lysis buffer and sonicated to fragment chromatin DNA. The 10% lysis sonicated chromatin lysate was stored as “input” sample; 80% was used in immunoprecipitation reactions with anti‐GFP antibody (Sigma) or anti‐RFP (EnoGene, E12‐037) and named “IP”; and 10% was incubated with mouse IgG (CST) as a negative control and named “IgG.” The DNA from input and IP samples was extracted by the phenol‐chloroform method. High‐throughput DNA sequencing libraries were prepared using VAHTS Universal DNA Library Prep Kit for Illumina V3 (Vazyme, ND607). Library products corresponding to 200–500 bps were enriched, quantified, and finally sequenced on Novaseq 6000 sequencer (Illumina) in PE150 mode.

Raw sequencing data were first filtered using Trimmomatic (v0.36). Low‐quality reads were discarded, and reads contaminated with adaptor sequences were trimmed. The clean reads were used for protein‐binding site analysis. They were mapped to the potato reference genome from PGSC (https://spuddb.uga.edu/dm_v6_1_download.shtml) using STAR software (v2.5.3a) with default parameters. RSeQC (version 2.6) was used for read distribution analysis. MACS2 software (v2.1.1) was used for peak calling. Bedtools (v2.25.0) was used for peak annotation and peak distribution analysis. Differentially bound peaks were identified using a Python script with the Fisher's test. HOMER (v4.10) was used for motif analysis. Gene Ontology (GO) analysis and Kyoto Encyclopedia of Genes and Genomes (KEGG) enrichment analysis for annotated genes were both performed using KOBAS software (v2.1.1), with a corrected *P*‐value cutoff of 0.05 to judge statistically significant enrichment.

### RNA Seq and data analysis

RNA‐seq experiments, high‐throughput sequencing, and data analysis were conducted by Seqhealth Technology Co., Ltd (Wuhan, China). Two transgenic potato lines overexpressing *StKNOX3*, two transgenic potato lines silencing *StKNOX3*, together with the wild‐type “E3,” were collected for total RNA extraction. Total RNA from three biological replicates was extracted using TRIzol reagent (ZOMANBIO). RNA integrity was confirmed by 1.5% agarose gel electrophoresis.

Raw sequencing data were first filtered using Trimmomatic (version 0.36); low‐quality reads were discarded, and reads contaminated with adaptor sequences were trimmed. Clean reads were further processed with in‐house scripts to eliminate duplication bias introduced during library preparation and sequencing. Briefly, clean reads were first clustered according to unique molecular identifier (UMI) sequences, where reads sharing the same UMI sequence were grouped into the same cluster. Reads in the same cluster were compared by pairwise alignment, and then reads with a sequence identity above 95% were extracted to a new subcluster. After all subclusters were generated, multiple sequence alignment was performed to obtain one consensus sequence for each subcluster. Following these steps, errors and biases introduced by PCR amplification or sequencing were eliminated.

The de‐duplicated consensus sequences were used for standard RNA‐seq analysis. They were mapped to the potato reference genome from PGSC (DM 1‐3 516 R44 Genome Assembly and Annotation (v6.1)) using STAR software (v2.5.3a) with default parameters. Reads mapped to the exon regions of each gene were counted by featureCounts (Subread‐1.5.1; Bioconductor), and then, RPKM values were subsequently calculated. Genes differentially expressed between groups were identified using the edgeR package (v3.12.1). Statistical significance of gene expression differences was determined using a *P*‐value cutoff of 0.05 and fold‐change cutoffs of 1.5 (|log_2_| ≥ 0.6) for *StKNOX3* samples. Gene Ontology (GO) analysis and Kyoto Encyclopedia of Genes and Genomes (KEGG) enrichment analysis for differentially expressed genes were both performed using KOBAS software (v2.1.1) with a *P*‐value cutoff of 0.05 to determine statistically significant enrichment. Alternative splicing events were detected using rMATS (v3.2.5) with a false discovery rate (FDR) value cutoff of 0.05 and an absolute value of Δψ of 0.05.

### Electrophoretic mobility shift assay (EMSA)

The coding sequence of *StKNOX3* and *Pi22798* was cloned into the expression vector pET32a, which was transformed into *Escherichia coli* (*E. coli*) strain Rosetta (DE3). The fused proteins (His‐StKNOX3 and His‐Pi22798) were induced by 0.5 mM isopropyl‐β‐D‐1‐thiogalactopyranoside (IPTG) and purified using Ni‐NTA Agarose according to the manufacturer's instructions. The DNA probes containing an original TGTCA or TGAC motif, or mutated sequence, with flanking sequence were synthesized as FAM‐labeled probes at Sangon Biotech (Beijing, China). A 10 × excess of unlabeled probes was used as a competitor. Purified His‐tagged proteins were incubated with FAM‐labeled probes at room temperature for 15 min with or without competitors (unlabeled probes or mutant probes). The resulting reaction mixtures were subjected to 5% non‐denaturing PAGE in 0.5 × TBE buffer and were imaged with a Multicolor Fluorescence and Chemiluminescence Imaging System (Bio‐Rad).

### Dual LUC assay

The plasmids containing StKNOX3‐RFP under the control of the CaMV 35S promoter were used as effectors. For the reporters, the promoters of *StLTP2* and *StH2A.Z* were cloned upstream of the firefly LUC reporter gene into the pGREEN II‐0800‐LUC vector, which also contains the Renilla LUC (REN) gene driven by the CaMV 35S promoter. Effectors and reporters were individually transformed into *Agrobacterium* strain GV3101 containing the helper plasmid pSoup‐P19, then co‐infiltrated into *N. benthamiana* leaves. LUC and REN activities were measured with a dual luciferase reporter gene assay kit (Yeasen) according to the manufacturer's instructions using a microplate reader (Roche). The relative LUC activity was calculated as the ratio of LUC to REN activity. Three biological replicates were used for each effector–reporter combination.

### Statistical analysis

All data in this study were analyzed using GraphPad Prism 8. A two‐tailed Student's *t*‐test or two‐way analysis of variance (ANOVA) with Tukey's test was used for pairwise or multiple comparisons, respectively. Statistical significance was determined using these tests. All values and error bars presented are means ± SEM of three or more experimental replicates.

## AUTHOR CONTRIBUTIONS

ZT and JZ planned and designed the research. JZ, XZ, JN, YQ, XW, LL, RY, and LG performed the experiments. YZ provided help for data analysis. PRJB provided help for writing. JZ and ZT analyzed data and wrote and revised the manuscript.

## CONFLICT OF INTEREST

The authors have no conflicts to declare.

## Supporting information


**Figure S1.** Alignment and phylogenetic tree of HUB1/2 from potato, *Arabidopsis*, *Oryza sativa*, and *Nicotiana benthamiana*.
**Figure S2.** Expression of spilt‐LUC interaction constructs in *Nicotiana benthamiana*.
**Figure S3.** Sub‐localization of GFP‐StHUB1 and GFP‐StHUB2 and their colocalization with StKNOX3‐RFP or RFP‐StKH17 in *Nicotiana benthamiana* cells.
**Figure S4.** Expression of BiFC interaction constructs in *Nicotiana benthamiana*.
**Figure S5.** Changes of expression of *StHUB1* and *StHUB2* during *Phytophthora infestans* infection and gene expression levels of *StHUB1* and *StHUB2* in transgenic potato plants.
**Figure S6.** Sub‐localization of GFP‐_NLS_StHUB1 and GFP‐_NLS_StHUB2.
**Figure S7.** NLS mutant of Pi22798 compromises its virulence.
**Figure S8.** Overexpressing *StKNOX3* reduces resistance against *Phytophthora infestans* in potato “E3” variety.
**Figure S9.** Analysis of StKNOX3‐binding sites in potato genome.
**Figure S10.** Silencing *StKNOX3* enhances resistance against *Phytophthora infestans* in potato “E3”.
**Figure S11.** Identification of StLTP2 and StH2A.Z homologs in potato by phylogenetic analysis through BLAST search.
**Figure S12.** Expression of StLTP2‐HA and StH2A.Z‐HA constructs in *Nicotiana benthamiana*.

**Data S1.** Interacting proteins of StKNOX3 by Y2H screening.

**Data S2.** List of StKNOX3‐binding sites identified by ChIP‐Seq.

**Data S3.** List of differentially expressed genes (DEGs) identified in *StKNOX3*‐OE potato plants using RNA‐Seq.

**Data S4.** GO analysis of targeted DEGs in wild‐type and *StKNOX*3‐OE potato plants.

**Data S5.** Primers used to construct vectors and qRT‐PCR.

## Data Availability

All data supporting the findings of this study are available in the main text or Supplementary Information. The accession numbers of proteins used in this study are as follows: StKNOX3 (PGSC0003DMT400078989), StHUB1 (XM_006366868.2), StHUB2 (XP_006354162.1), StKH17 (XM_006362273.2), Pi22798 (XP_002998395.1). Source data are provided in this paper. All raw and processed sequencing data have been deposited in the NCBI Sequence Read Archive (SRA). The accession ID numbers are as follows: RNA‐seq data: PRJNA1503056 and ChIP‐seq data: PRJNA1499296.
