# Supplementary material for: A Phytophthora effector hijacks host StKNOX3‐StHUB1/2 complex to reprogram defense gene expression via histone monoubiquitination
Source: Plant J. 2026 Sep 14;127(5):e71112. doi: 10.1111/tpj.71112 (PMC13575310; doi:10.1111/tpj.71112)
Supplement: Supplementary file 1 [file TPJ-127-0-s002.docx]

**SUPPORTING INFORMATION**

**Figure S1.** Alignment and phylogenetic tree of HUB1/2 from potato, *Arabidopsis*, *Oryza sativa* and *N. benthamiana*.

**Figure S2.** Expression of spilt-LUC interaction constructs in *N. benthamiana*

**Figure S3.** Sub-localization of GFP-StHUB1 and GFP-StHUB2, and their colocalization with StKNOX3-RFP or RFP-StKH17 in *N. benthamiana* cells.

**Figure S4.** Expression of BiFC interaction constructs in *N. benthamiana.*

**Figure S5.** Changes of expression of *StHUB1* and *StHUB2* during *P. infestans* infection and gene expression levels of *StHUB1* and *StHUB2* in transgenic potato plants.

**Figure S6.** Sub-localization of GFP-_NLS_StHUB1 and GFP-_NLS_StHUB2.

**Figure S7.** NLS mutant of Pi22798 compromises its virulence.

**Figure S8.** Overexpressing *StKNOX3* reduces resistance against *P. infestans* in potato ‘E3’ variety.

**Figure S9.** Analysis StKNOX3 binding sites in potato genome.

**Figure S10.** Silencing *StKNOX3* enhances resistance against *P. infestans* in potato ‘E3’.

**Figure S11.** Identification of StLTP2 and StH2A.Z homologs in potato by phylogenetic analysis through BLAST search.

**Figure S12.** Expression of StLTP2-HA and StH2A.Z-HA constructs in *N. benthamiana.*

**Data S1.** Interacting proteins of StKNOX3 by Y2H screening.

**Data S2.** List of StKNOX3 binding sites identified by ChIP-Seq.

**Data S3.** List of differentially expressed genes (DEGs) identified in *StKNOX3*-OE potato plants using RNA-Seq.

**Data S4.** GO analysis of targeted DEGs in wild type and *StKNOX*3-OE potato plants.

**Data S5.** Primers used to construct vectors and qRT-PCR.

**Figure S1.** Alignment and phylogenetic tree of HUB1 and HUB2 from potato, *Arabidopsis*, *Oryza sativa* and *N. benthamiana*.

(a) Amino acid sequence alignment of HUB1 and HUB2 from *Solanum lycopersicum*, *Solanum tuberosum*, *Nicotiana benthamiana* and *Arabidopsis thaliana*, respectively*.*

(b) Phylogenetic tree of HUB1 and HUB2 protein sequences was constructed using the Neighbor‑Joining method with the MEGA X program.

**Figure S2.** Expression of spilt-LUC interaction constructs in *N. benthamiana.*

Protein was extracted 2-3 days post-ago-infiltration. Luciferase antibody was used for immunoblots. Red asterisks show the presence of the target protein bands. Protein sizes are indicated by kDa. The predicated molecular weights of EV-nLUC, StHUB1-nLUC, StHUB2-nLUC, cLUC-RFP, and cLUC-StKNOX3 are 45 kDa, 145 kDa, 150 kDa, 42 kDa, and 75 kDa, respectively.

**Figure S3.** Sub-localization of GFP-StHUB1 and GFP-StHUB2, and their colocalization with StKNOX3-RFP or RFP-StKH17 in *N. benthamiana* cells.

(a) Both GFP-StHUB1 and GFP-StHUB2 fusion proteins localized at cytoplasm and nucleus in transiently infiltrated *N. benthamiana* leaves. Scale bars, 25 μm.

(b) Western blot showing the protein stabilities of GFP, GFP-StHUB1 and GFP-StHUB2 detected using an anti-GFP antibody. Protein sizes are indicated by kDa. Target protein bands are marked with red asterisks.

(c-d) Co-localization of StKNOX3-RFP, RFP-StKH17 with GFP-StHUB1 (c) or GFP-StHUB2 (d) in transiently infiltrated *N. benthamiana* leaves, respectively. Scale bars, 50 μm.

**Figure S4.** Expression of BiFC interaction constructs in *N. benthamiana*.

Protein was extracted 2-3 days post ago-infiltration and fusion proteins shown to be stable. HA or cMyc antibody was used for immunoblots of YFP^C^ and YFP^N^ construct, respectively. Protein sizes are indicated by kDa. Red asterisks show the presence of the target protein bands. The predicated molecular weights of YFP^N^-RFP, YFP^N^-StHUB1, YFP^N^-StHUB2, and StKNOX3-YFP^C^ are 40 kDa, 125 kDa, 135 kDa, and 75 kDa, respectively.

**Figure S5.** Changes in expression of *StHUB1* and *StHUB2* during *P. infestans* infection and gene expression levels of *StHUB1* and *StHUB2* in transgenic potato plants.

(a-b) Expression of *StHUB1* and *StHUB2* in leaves of the wild-type (E3) during *P. infestans* infection, as determined by qRT-PCR. Expression is relative to that of *Tubulin* (*Tub*). Data are means ± SEM of three replicates.

(c) Expression of *StHUB1* and *StHUB2* in leaves of the wild-type (E3) and overexpression potato lines, as determined by qRT-PCR. Expression is relative to that of *Tubulin* (*Tub*). Data are means (± SEM) of three replicates.

(d) Expression of *StHUB1* and *StHUB2* in leaves of the wild-type (E3) and dual silencing potato lines as determined by qRT-PCR. Expression is relative to that of *Tubulin* (*Tub*). Data are presented as means ± SEM of three replicates.

(e) Silencing *StHUBs* enhances *P. infestans* infection in potato. Data represent three biological repeats (One-way ANOVA, ** *P* < 0.01, ns, no significant difference).

**Figure S6.** Sub-localization of GFP-_NLS_StHUB1 and GFP-_NLS_StHUB2.

(a-b) Confocal images show that GFP-_NLS_StHUB1 and GFP-_NLS_StHUB2 cause StHUB1 and StHUB2 to be localized in the nucleus, respectively. Both GFP-fusion proteins localized in the nucleus in transiently infiltrated *N. benthamiana* leaves. Scale bars are as indicated.

(c) Expression of constructs EV-GFP, GFP-StHUB1, GFP-_NLS_StHUB1, GFP-StHUB2, and GFP-_NLS_StHUB2 in *N. benthamiana*. Protein was extracted 3 days post-ago-infiltration. GFP antibody was used for immunoblots. Protein sizes are indicated by kDa. Target protein bands are indicated by red asterisks. The predicated molecular weights of EV-GFP, GFP-StHUB1, GFP-_NLS_StHUB1, GFP-StHUB2, and GFP-_NLS_StHUB2 are 27 kDa, 128 kDa, 130 kDa, 132 kDa, and 134 kDa, respectively.

**Figure S7.** The NLS mutant of Pi22798 compromises its virulence.

(a) Schematic diagram of Pi22798 showing its nuclear localization signal (NLS) and the NLS mutant Pi22798_mNLS_.

(b) Representative images of subcellular localization of GFP alone, GFP-Pi22798 and the NLS mutant of Pi22798 in transiently expressed *N. benthamiana* leaves. Scale bars, 50 μm.

(c) *P. infestans* colonization of *N. benthamiana* increased significantly following *Agrobacterium*-mediated expression of GFP-Pi22798 compared with free GFP, but not following expression of GFP-Pi22798_mNLS_ (Multiple student’s *t-*test, * *P* < 0.05, three biological repeats).

(d) The Pi22798_mNLS_ mutant showed compromised interaction with StKNOX3 *in vivo*. Protein sizes are indicated by kDa. Target protein bands are indicated by red asterisks. The predicated molecular weights of EV-GFP, GFP-Pi22798, GFP-Pi22798_mNLS_ and Myc-StKNOX3-RFP are 27 kDa, 45 kDa, 45 kDa and 80 kDa, respectively.

(e) Expression of constructs EV-RFP, RFP-Pi22798, and RFP-Pi22798_mNLS_ in *N. benthamiana*. Protein was extracted 3 days post-ago-infiltration. GFP antibody was used for immunoblots. Red stars show the presence of the target protein bands. Protein sizes are indicated by kDa. The predicated molecular weights of RFP-Myc, RFP-Pi22798, and RFP-Pi22798_mNLS_ are 40 kDa, 45 kDa, and 45 kDa, respectively.

**Figure S8.** Overexpressing *StKNOX3* reduces potato resistance against *P. infestans*.

(a) Expression of *StKNOX3* in leaves of the wild-type (E3) and *StKNOX3-RFP*-OE potato lines, as determined by qRT-PCR. Expression is relative to that of *Tubulin* (*Tub*).

(b) Western blot showing the protein levels of StKNOX3-RFP in transgenic potato lines detected using anti-RFP antibody. Protein sizes are indicated by kDa. Targeted protein band is indicated by red asterisk. The predicated molecular weight of StKNOX3-RFP is 80 kDa.

(c-e) Graphs show a significant increase in lesion diameter (d) and spore numbers (e) in *StKNOX3-RFP*-OE transgenic potato lines compared with wild-type plants. The representative leaves indicate greater colonization on transgenic plants compared with control plants (one-way ANOVA; *** *P* < 0.001; **** *P* < 0.0001; three biological replicates).

**Figure S9.** Analysis of StKNOX3 binding sites in the potato genome.

(a) Percentage of reads distribution across different genomic regions. Promoter_2-1 kb: from 2 kb to 1 kb upstream of the transcription start site (TSS); Promoter_1 kb: 1 kb upstream of TSS; Downstream_2 kb: 2 kb downstream of TSS.

(b) Distribution of peaks in functional regions of genome. Exon and intron are corresponding to the gene models in genome annotation. Intergenic: gene-free region from 1,000-bp downstream of TES to 2,000-bp upstream of the TSS of the closest gene. Promoter-TSS: ± 2,000 bp of TSS.

(c) Distribution of reads near TSS.

(d) Heat maps showing the ChIP-Seq distribution of reads across TSS.

**Figure S10.** Silencing *StKNOX3* enhances potato resistance against *P. infestans*.

(a) Expression of *StKNOX3* in leaves of the wild-type (E3) and *StKNOX3*-silencing potato lines, as determined by qRT-PCR. Expression is relative to that of *Tubulin* (*Tub*).

(b,c) Graphs show a significant decrease of lesion diameter in transgenic potato lines silencing *StKNOX3* compared with wild-type plants. The representative leaves indicate less pathogen colonization on transgenic plants compared with the control plants (one-way ANOVA; **** *P* < 0.0001; three biological repeats). The data are represented as a box–whisker plot.

**Figure S11.** Identification of *StLTP2* and *StH2A.Z* homologs in potato by BLAST search (http://blast.jcvi.org/er-blast/).

(a,b) The phylogenetic tree is presented to scale, and branch lengths are measured in units of the evolutionary distances used for inference. The red arrows marked *StLTP2* and *StH2A.Z* studied in this work.

**Figure S12.** Expression of StLTP2-HA and StH2A.Z-HA constructs in *N. benthamiana*.

Protein was extracted 2 days post ago-infiltration and west blotting showed that fusion proteins were stably expressed. HA antibody was used for immunoblots. Protein sizes are indicated by kDa. Red asterisks show the presence of the target protein bands. The predicated molecular weights of HA-GUS, StLTP2-HA and StH2A.Z-HA are 80 kDa, 20 kDa, and 25 kDa, respectively.
